# Mutant HSPB1 causes loss of translational repression by binding to PCBP1, an RNA binding protein with a possible role in neurodegenerative disease

**DOI:** 10.1186/s40478-016-0407-3

**Published:** 2017-01-11

**Authors:** Thomas Geuens, Vicky De Winter, Nicholas Rajan, Tilmann Achsel, Ligia Mateiu, Leonardo Almeida-Souza, Bob Asselbergh, Delphine Bouhy, Michaela Auer-Grumbach, Claudia Bagni, Vincent Timmerman

**Affiliations:** 1 Peripheral Neuropathy Group, Department of Molecular Genetics, VIB, Institute Born Bunge and University of Antwerp, Antwerpen, Belgium; 2 Center for Human Genetics, Center for the Biology of Disease, VIB and KU Leuven, Leuven, Belgium; 3 Department of Fundamental Neuroscience (DNF), University of Lausanne, Lausanne, Switzerland; 4 Department of Molecular Genetics, VIB and University of Antwerp, Antwerpen, Belgium; 5 Present address: MRC Laboratory of Molecular Biology, Francis Crick Avenue, Cambridge, CB2 0QH UK; 6 Institute of Medical Biology and Department of Internal Medicine, Diabetes and Metabolism, Medical University Graz, Graz, Austria

**Keywords:** HSPB1, RNA immunoprecipitation, PCBP1, Distal Hereditary Motor Neuropathy, Charcot-Marie-Tooth

## Abstract

The small heat shock protein HSPB1 (Hsp27) is an ubiquitously expressed molecular chaperone able to regulate various cellular functions like actin dynamics, oxidative stress regulation and anti-apoptosis. So far disease causing mutations in HSPB1 have been associated with neurodegenerative diseases such as distal hereditary motor neuropathy, Charcot-Marie-Tooth disease and amyotrophic lateral sclerosis. Most mutations in HSPB1 target its highly conserved α-crystallin domain, while other mutations affect the C- or N-terminal regions or its promotor. Mutations inside the α-crystallin domain have been shown to enhance the chaperone activity of HSPB1 and increase the binding to client proteins. However, the HSPB1-P182L mutation, located outside and downstream of the α-crystallin domain, behaves differently. This specific HSPB1 mutation results in a severe neuropathy phenotype affecting exclusively the motor neurons of the peripheral nervous system. We identified that the HSPB1-P182L mutant protein has a specifically increased interaction with the RNA binding protein poly(C)binding protein 1 (PCBP1) and results in a reduction of its translational repressive activity. RNA immunoprecipitation followed by RNA sequencing on mouse brain lead to the identification of PCBP1 mRNA targets. These targets contain larger 3′- and 5′-UTRs than average and are enriched in an RNA motif consisting of the CTCCTCCTCCTCC consensus sequence. Interestingly, next to the clear presence of neuronal transcripts among the identified PCBP1 targets we identified known genes associated with hereditary peripheral neuropathies and hereditary spastic paraplegias. We therefore conclude that HSPB1 can mediate translational repression through interaction with an RNA binding protein further supporting its role in neurodegenerative disease.

**Electronic supplementary material:**

The online version of this article (doi:10.1186/s40478-016-0407-3) contains supplementary material, which is available to authorized users.

## Introduction

HSPB1 (Hsp27) is a member of the small heat shock protein family (sHSPs), comprising ubiquitously expressed molecular chaperones whose canonical function is to preserve cellular proteostasis during stress conditions. In contrast to heat shock proteins with an ATPase domain (e.g., HSP70), sHSPs do not have the intrinsic capacity to refold denatured proteins. However, they are able to bind to unfolded proteins keeping them in a folding-competent state until they are properly refolded by the ATP-dependent heat shock proteins [17]. Besides this canonical function, HSPB1 is also known to regulate actin dynamics, cell differentiation and to have anti-apoptotic and anti-oxidant functions [5]. HSPB1 is characterized by a highly conserved α-crystallin domain, which is found essential for its biological function and dimer formation [17].

So far, 26 mutations (including 22 missense mutations) have been found in HSPB1 that are responsible for axonal Charcot-Marie-Tooth neuropathy (CMT2F), distal hereditary motor neuropathy (dHMN) and amyothropic lateral sclerosis (ALS) [7, 11, 14, 27, 49]. Depending on where the mutations are located in the *HSPB1* gene, they can have different consequences on the protein function and patient’s clinical outcome. The vast majority of mutations found in HSPB1 are linked with CMT2F/dHMN type II and reside in the well-conserved α-crystallin domain [37]. The CMT2F patients typically present with mixed sensory and motor symptoms, while motor neurons are predominantly affected in patients with dHMN type II [24]. It is surprising that mutations in this ubiquitously expressed molecular chaperone specifically affect the peripheral nerves suggesting a key role in the highly polarized motor and sensory neurons.

In this work we specifically focus on the P182L mutation in HSPB1 which was first reported in 2004 in a sibling of an Austrian dHMN family [14]. The onset of the disease phenotype was at the age of 5 and although the last examination was at the age of 16, there was already the presence of gait difficulties and weakness of the distal upper and lower limbs associated with muscular atrophy. The formation of *pes cavus* and the manifestation of brisk knee reflexes were also reported [11]. Interestingly, when studying the functional consequences of this particular HSPB1 mutation, the severity of the P182L mutation was further supported by an increased presence of aggregates in cell lines upon overexpression of the mutant protein [1]. In addition, and similar to the other studied HSPB1 mutations, the P182L mutation causes an increase in the phosphorylation of neurofilaments and disturbances in the axonal transport [20]. Whereas the CMT2F causing HSPB1 mutations in the α-crystallin domain of the protein show an increased binding to their client proteins leading to an overstabilization of microtubules, the P182L mutation does not cause this aberration and behaves as the wild-type HSPB1 protein [3, 4]. Overall, this indicates that the increased clinical severity of the P182L mutation is caused by additional, unidentified factors.

In order to characterize HSPB1 mutations we previously performed interaction studies by using tandem affinity purification coupled to mass spectrometry (TAP-MS) [4]. This study revealed an RNA binding protein named poly(C)binding protein1 (PCBP1) as an unreported and novel interaction partner for the wild type and P182L mutant HSPB1. Here, we studied the interaction between PCBP1 and wild type HSPB1 through in vitro experiments and found the interaction to be increased for HSPB1-P182L, both in a dHMN patient derived lymphoblastoid cell line and in HeLa cells transiently expressing wild type and mutant HSPB1. Interestingly, this increased interaction resulted in a loss of translational repression of PCBP1 on its mRNA targets. By using RNA immunoprecipitation followed by RNA sequencing we identified the targets that specifically bind to PCBP1. Of note, there are nine genes among these targets that when mutated are known causes for inherited peripheral neuropathies (IPN) and hereditary spastic paraplegia (HSP) (*Inf2, Sox10, Wnk1, Med25, Fa2h, Bscl2, Slc12a6, Kif1a* and *Kif5a*) [13, 21, 29, 31, 40–42, 52].

## Materials and methods

### Clinical phenotype of dHMN patients with the HSPB1-P182L mutation

The first reported patient in 2004 carrying an HSPB1-P182L mutation (c.545C > T), patient CMT-391:II.I, belonged to an Austrian family where also a younger sibling was affected. A disease phenotype was reported at the age of 5 and until the last examination at the age of 16, the patient showed gait difficulties, weakness of the distal upper and lower limbs associated with muscular atrophy. In addition, *pes cavus* and brisk knee reflexes were reported. None of the asymptomatic parents showed the heterozygous HSPB1-P182L mutation but a parental mosaicism for the mutation was identified [11]. This patient was clinically diagnosed by M.A.G.

### Creation of constructs and stable cell lines

Constructs used for transient transfection experiments and to generate stable SH-SY5Y cell lines were designed using the Gateway recombination system (Life Technologies). The open reading frames (ORF) of HSPB1 (NM_001540), PCBP1 (NM_006196) and EGFP were amplified by PCR using specific primers flanked by attB recombination sites to allow the insertion of the product in the pDONR221 vector. The described HSPB1 mutations (HSPB1-R127W and HSPB1-P182L) were generated by site-directed mutagenesis. Sequence validated pDONRs were transferred by recombination to the pLenti6/V5 destination vector (Life Technologies) allowing us to generate constructs where the ORF is fused to a V5-tag. To generate the PCBP1-VSV construct, the pDONR was transferred by recombination to a pCR3/VSV destination vector. For the luciferase assays we cloned the PCBP1 and EGFP cDNA’s upstream of a MS2 binding protein ORF containing plasmid [50]. All the final plasmids were verified by Sanger sequencing.

Stable cell lines were generated by lentiviral transduction of the neuronal cell line SH-SY5Y, according to the method described previously [43]. Stable cell lines presented similar expression levels and equal growth rate (data not shown).

### Cell culture material and conditions

All cell culture media and supplements were purchased from Life Technologies. The human neuroblastoma cell line SH-SY5Y was purchased from ATCC and cultivated at 37 °C and 5% CO_2_ in EMEM supplemented with 10% fetal calf serum (FCS), non-essential amino acids, Glutamine and Penicillin-Streptomycin. The HeLa cell line was purchased from ATCC and cultivated at 37 °C and 5% CO_2_ in DMEM supplemented with 10% FCS, Glutamine and Penicillin-Streptomycin. The HEK293 cell line was purchased from ATCC and cultivated at 37 °C and 5% CO_2_ in DMEM supplemented with 10% FCS, HEPES, Glutamine and Penicillin-Streptomycin. The NSC-34 cell line was purchased from ATCC and cultivated at 37 °C and 5% CO_2_ in DMEM supplemented with 10% FCS, HEPES, Glutamine and Penicillin-Streptomycin. From the patients we obtained lymphocytes through venipuncture and lymphoblastoid cell lines were EBV-transformed and cultivated at 37 °C and 6% CO_2_ in Gibco® RPMI 1640 supplemented with 15% FCS, Sodium Pyruvate and Penicillin-Streptomycin. Besides the described HSPB1-P182L patient we also obtained lymphocytes from an HSPB1-R127W patient (cmt751.01) and a healthy control individual (ceph1454.14). Fibroblasts were obtained from patients by taking skin biopsies and cultivated at 37 °C and 6% CO_2_ in DMEM/F12 supplemented with 10% FCS, Sodium Pyruvate and Penicillin-Streptomycin. We obtained approval from our local medical ethical committees to perform this study.

### Transient transfection

HeLa cell lines were transfected by using Lipofectamine LTX transfection reagent (Life Technologies) according to the manufacturer’s protocol. Briefly, cells were seeded out in 10 cm dishes at 1.5 × 10^6^ cells per dish 24 h before transfection. On the day of transfection, media was renewed without the addition of antibiotics. Transfection was performed using 4000 ng plasmid DNA supplemented with 20 μl Lipofectamine LTX transfection reagent. Cells were lysed and assessed by co-immunoprecipitation, 24 h post transfection.

HEK293 cell lines were transfected by using polyethylenimine (PEI) according to an in house optimized protocol. Briefly, cells were seeded out in a 24 well plate at 7x10^4^ cells per well, 24 h before transfection. On the day of transfection, media was renewed without the addition of antibiotics and containing a reduced serum concentration (2%). 500 ng plasmid DNA was diluted in 36 μl Opti-MEM (Life-Technologies) and in parallel, 2.5 μl PEI was diluted in 36 μl Opti-MEM. The diluted PEI was added to the DNA and mixed by gently vortexing. After 15 min of incubation at room temperature, the solution was added to the cells.

### Western blotting

Cells were lysed in lysis buffer (0.5% Nonidet P-40, 137 mM NaCl, 2.7 mM KCl, 10 mM Na_2_HPO_4_, 1.8 mM KH_2_PO_4_, 4 mM Sodium orthovanadate, 20 mM Glycerol-2-Phosphate, 10 mM Sodium Fluoride, 1 mM Sodium Pyrophosphate, together with complete protease and Phospho-STOP inhibitor mixtures - Roche Applied Science) for 30 min on ice and cleared by centrifugation for 10 min at 14,000 rpm. After protein concentration was determined, by using the Bradford protein assay (Bio-Rad), cell lysates were boiled for 5 min at 95 °C in reducing Laemmli buffer (Life Technologies) supplemented with 100 mM 1.4-Dithiothreitol (DTT). Proteins were separated on NuPAGE gels (Life Technologies) and transferred to a nitro-cellulose-membrane (Hybond™-P, GE Healthcare). Blocking of the membrane was performed using 5% milk powder diluted in PBS, supplemented with 0.1% Tween 20. Afterwards, membranes were incubated with a primary antibody over night at 4 °C and one hour with a secondary horseradish peroxidase conjugated antibody. Blots were developed by using the Enhanced Chemiluminescence ECL Plus™ detection system (Thermoscientific) and imaged with an ImageQuant imager (GE Healthcare). The following antibodies were used: anti-HSPB1 (Enzo Life Sciences), anti-V5 (Life Technologies), anti-VSV (Sigma-Aldrich) and anti-PCBP1 (Santa Cruz Biotechnology and Novus Biologicals).

Band intensities were determined by quantifying the mean pixel grey values using the ImageJ software [44]. Mean pixel grey values were measured in a rectangular region of interest.

### Co-immunoprecipitation

Cells were lysed in lysis buffer (0.5% Nonidet P-40, 137 mM NaCl, 2.7 mM KCl, 10 mM Na_2_HPO_4_, 1.8 mM KH_2_PO_4_, 4 mM Sodium orthovanadate, 20 mM Glycerol-2-Phosphate, 10 mM Sodium Fluoride, 1 mM Sodium Pyrophosphate, together with complete protease and Phospho-STOP inhibitor mixtures - Roche Applied Science) for 30 min on ice and cleared by centrifugation for 10 min at 14,000 rpm. Protein concentration was determined, by using the Bradford protein assay (Bio-Rad), in order to equalize the protein concentration from different cell lines. Sepharose 6B (Sigma-Aldrich) and protein G beads (GE Healthcare) or anti-V5 beads (Sigma-Aldrich) were used in a 3:1 ratio. Cell lysates with an equal protein concentration were incubated together with the bead mixture and the desired antibody, when needed, and left on a rotating device overnight at 4 °C. Afterwards the beads were collected by centrifugation and washed repeatedly with the same buffer as used for lysis of the cells. After the last washing step beads were resuspended in reducing Laemmli buffer (Life Technologies) supplemented with 100 mM DTT and boiled for 5 min at 95 °C prior to be loaded on NuPAGE gels (Life Technologies).

### Polysome Gradient Analysis

Polysomal and non-polysomal fractions were separated according to a standardized protocol. Briefly, cells were collected by centrifugation, lysed in lysis buffer (30 mM Tris-HCl; pH7.4, 100 mM NaCl, 10 mM MgCl_2_ and 0.1% Triton X-100) and clarified by centrifugation for 10 min at 10.000 × g. The supernatant was transferred onto a 5–50% sucrose gradient (30 mM Tris-HCl; pH7.4, 100 mM NaCl, 10 mM MgCl_2_ and 5–50% sucrose) and centrifuged at 37,000 rpm for 3.5 h at 4 °C in a Beckman-Coulter LE-80 K. Fractions of each gradient were collected by monitoring at 260 nm and precipitated with 50% ethanol, 25% methanol and 25% acetone. After precipitation, pellets were washed in 0.3 M Guanidinium-HCl in 96% ethanol. The purified pellet was dissolved in Laemmli buffer (Life Technologies) supplemented with 100 mM DTT and boiled for 5 min at 95 °C prior to be loaded on NuPAGE gels (Life Technologies).

### Luciferase assay

HEK293 cells were co-transfected with a luciferase reporter, PCBP1 or EGFP and HSPB1 wild type or mutants by using PEI as described before. Luciferase activity was measured with the Dual-luciferase reporter assay system (Promega) and following the manufacturer’s protocol. The luciferase activity was normalized by the renilla activity in order to correct for inter-well variations.

### Immunocytochemistry and immunofluorescence microscopy

Immunostainings were performed according to a standardized protocol. Briefly, cells were seeded out on glass coverslips the day before fixation. Fixation was performed by incubating the cells with 4% paraformaldehyde for 20 min. Afterwards cells were permeabilized with 0.1% Triton X-100 in phosphate buffered saline (PBS). Blocking was performed with 5% BSA diluted in PBS for 1 h, primary and secondary antibodies were incubated for 1 h diluted in 1% BSA in PBS supplemented with 0.1% Tween 20. The following antibodies were used; anti-HSPB1 (Enzo Life Sciences), anti-V5 (Life Technologies), anti-NFH (Covance), anti-PCBP1 (Santa Cruz Biotechnology), anti-mouse IgG Alexa Fluor 488 (Life Technologies), anti-goat IgG Alexa Fluor 488 (Life Technologies), anti-mouse IgG Alexa Fluor 594 (Life Technologies) and anti-goat IgG Alexa Fluor 594 (Life Technologies). Nuclear staining was performed with DAPI (Life Technologies), afterwards cells were mounted with fluorescent mounting medium (Dako). Images were taken on a LSM700 confocal fluorescence microscope using a 63×/1.40 plan-apochromatic objective. Possible cross-talk of the fluorescence channels was excluded by employing frame-by-frame scanning.

### Neurite outgrowth of neuroblastoma cells

The human neuroblastoma cell line SH-SY5Y stably expressing different HSPB1 constructs was cultured in the presence of 20 μM retinoic acid (Sigma Aldrich) to induce neuronal differentiation after seeding. Phase contrast images were acquired with a Carl Zeiss Axiovert 200 M using a 10× objective (Plan NeoFluar Ph1 0.3 NA) and Zeiss AxioCamMR3 camera. Images were acquired of the same microscopic fields of cells on consecutive days, making use of the automated positioning function of the motorized microscope stage. To determine neurite outgrowth, a specific image analysis procedure was developed in ImageJ [44] to extract from each image the neuritic structures from the background and cell bodies. The pipeline consists of a combination of noise reduction filters, bandpass filtering in the Fourier space, local and global intensity thresholding, and combining resulting masks of different segmentation algorithms with Boolean operations. The final neurite mask was derived after a skeletonization step and its total size was used as a measure for the neurite length. The analysis was run in batch on all images using an ImageJ macro script.

### RNA isolation from mouse tissues

To measure the abundance of mRNA in various mouse tissues, RNA isolation was performed. Animals were euthanized by CO_2_ inhalation and the brain, heart, liver, lung, muscle, sciatic nerve and spinal cord were dissected out and snap frozen in liquid nitrogen. The frozen tissues were homogenized in Trizol (Qiagen) and total RNA was isolated by using the RNeasy Lipid Tissue Mini kit (Qiagen) according to manufacturer’s protocol. All mouse experiments were approved by the Ethical Committee for Laboratory Animals (University of Antwerp). The mice were housed and maintained at the Animal Facility Interfaculty Unit, which is accredited by the Association for Assessment and Accreditation of Laboratory Animals.

### RNA-immunoprecipitation (RNA-IP)

Total mouse brain was lysed in lysis buffer (50 mM Tris-HCl; pH 7.4, 150 mM NaCl, 0.1% Triton X-100, 1 mM DTT together with complete protease inhibitor mixture - Roche Applied Science), put on ice for 30 min and cleared by centrifugation for 10 min at 14,000 rpm. 40 Units/ml RNase OUT (Life Technologies) was added to the cleared lysate. After determining the protein concentration by using the Bradford protein assay (Bio-Rad), the lysate was diluted to a final concentration of 1 mg/ml in immunoprecipitation buffer (50 mM Tris-HCl; pH 7.4, 200 mM NaCl, 0.1% Triton X-100 and 2 mg/ml Heparin) and prepared for preclearing. Preclearing was performed on the diluted lysate by adding 50 μl Dynabeads (Life Technologies) without the presence of an antibody. After leaving the beads on a rotating device for 60 min at 4 °C, supernatant was taken for immunoprecipitation by adding 50 μl Dynabeads supplemented with 7 μg PCBP1 antibody (Santa Cruz Biotechnology). In parallel, a negative control was created by using 50 μl Dynabeads and 7 μg normal goat IgG (Santa Cruz Biotechnology) in order to check for background mRNAs binding to the Dynabeads. As for the preclearing, the samples for immunoprecipitation were left on a rotating device for 60 min at 4 °C. Afterwards beads were washed four times with washing buffer (50 mM Tris-HCl; pH 7.4, 200 mM NaCl, 0.1% Triton X-100 and 1 mM MgCl_2_). After the final wash beads were resuspended in elution buffer (200 mM NaOAc, 1 mM EDTA and 0.5% SDS) and heated up to 75 °C for 5 min. RNA was isolated on this fraction by phenol/chloroform extraction.

### RNA sequencing

Purified RNA originating from the RNA-IP was sequenced on an Illumina HiSeq platform by paired-end sequencing. Library prepping was performed by using the TruSeq RNA Library Prep Kit from Illumina. The sequencing data were mapped to the mouse mm10 reference genome (Release date: December 2011) and thoroughly analyzed. Differential gene expression analysis was performed with the DESeq algorithm (Bioconductor 2.13).

### RT-qPCR

Isolated RNA was assessed on concentration and purity by Nanodrop (Thermoscientific) measurement. DNase treatment was performed by using the TURBO™ DNAse treatment kit (Life Technologies) and cDNA conversion was performed by using the M-MLV Reverse Transcriptase system (Life Technologies) according to manufacturer’s protocol. To determine the amount of a transcript being present in the RNA-IP, relative expression levels were determined by qPCR experiments. The reactions were performed using Power SYBR® Master Mix (Life Technologies) according to manufacturer’s instructions and run on a ViiA 7 Real-Time PCR system (Applied Biosystems). All the reactions were preformed in triplicate and normalized to IgG as negative control. Primers were designed making use of Primerbank (http://pga.mgh.harvard.edu/primerbank). Primers were validated for specificity and amplification efficiency.

### Calculation motif frequencies

Biomart and Perl API from Ensembl was used to extract the genomic coordinates and the sequences for the exons, 5′UTRs, 3′UTRs and introns belonging to the coding transcripts in mouse database v82. Only the transcripts having complete annotation and sequences available for all 4 features: exons, 5′UTR, 3′UTR and introns were used. The motif frequency was calculated in each of the 4 features of each transcript using the count of the exact match for the motif CTCCTCCTCCTCC (and reverse complement; GAGGAGGAGGAGG) in the feature and normalized to the transcript length.

### Statistical analysis

For all experiments, results are shown as average with standard deviation. GraphPad Prism software was used for all statistical analysis. One way ANOVA with Holm-Sidak multiple comparison Test was used to analyze the quantification of the ratio PCBP1/HSPB1 in transiently transfected HeLa cell lines. To analyze the median 3′- and 5′-UTR length of the top 50 PCBP1-regulated targets a One Sample Wilcoxon signed-rank test was performed. Multiple *t*-test with Holm-Sidak as a correction for multiple comparisons was performed as statistical test for the qPCR validation graphs. The p-values were calculated on at least three independent experiments. A two-sample one-sided Kolmogorov-Smirnov test in R package was performed to test statistical significance in the motif frequencies analyzed in 3′-UTR, 5′-UTR, introns and exons of enriched target genes.

## Results

### Mutant HSPB1 shows an increased affinity for PCBP1, a novel binding partner

In order to address how mutations in HSPB1 may cause CMT neuropathy, we previously performed a tandem affinity purification on HEK293 cells stably expressing HSPB1 wild type and different CMT causing mutations [4]. Mass spectrometry analysis resulted in the identification of the poly(C)binding protein 1 (PCBP1) as an interacting protein for wild type HSPB1 showing an increased affinity for mutant HSPB1. PCBP1 has been catalogued as an RNA binding protein and alternatively named as heterogeneous nuclear protein E1 (hnRNP E1). Together with HSPB1, PCBP1 is ubiquitously expressed and present in mouse peripheral nervous tissues (Additional file 1: Figure S1).

**Fig. 1:**
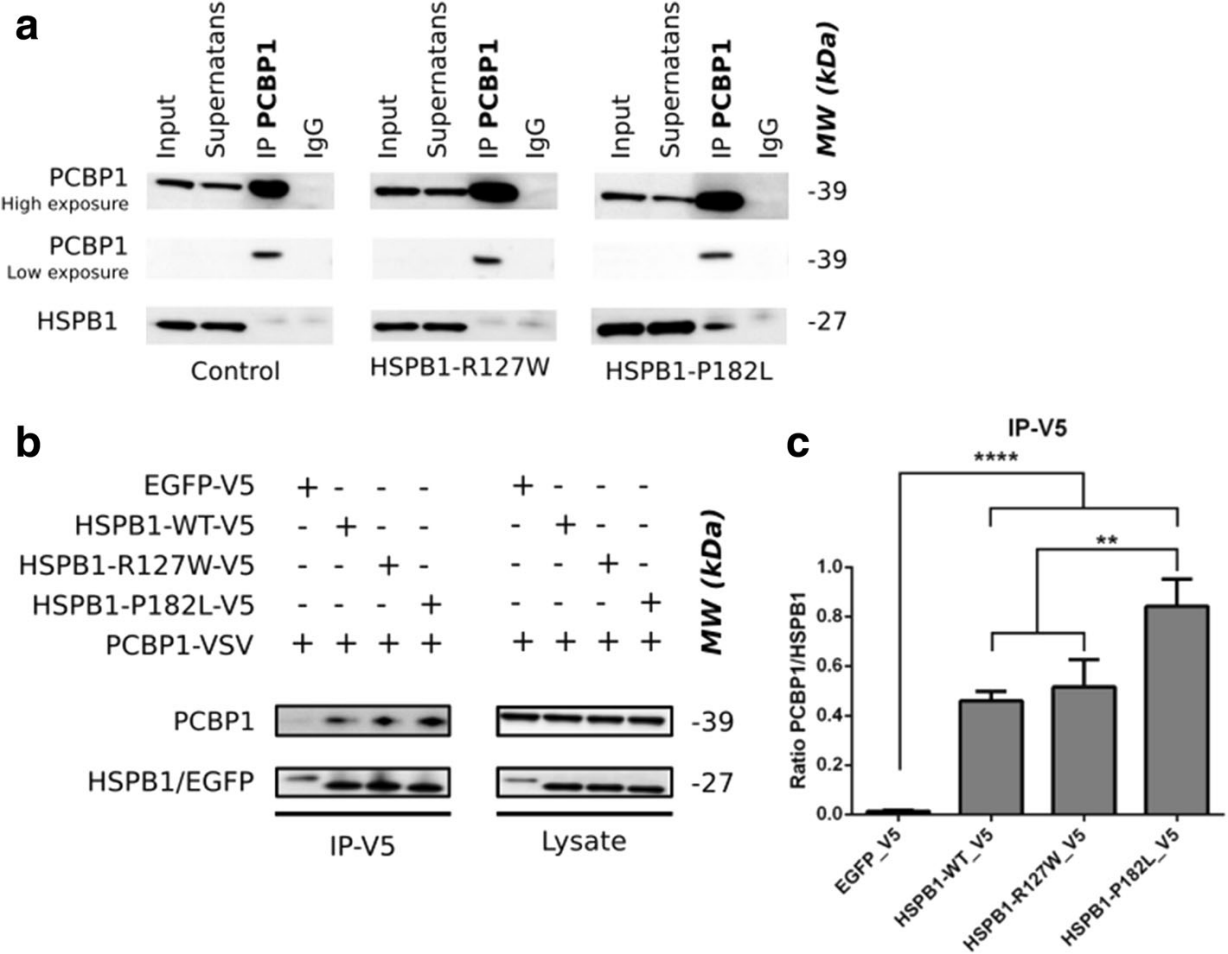
Identification of PCBP1 as a novel interaction partner of wild type and mutant HSPB1. **a** Co-immunoprecipitation was performed on patient-derived lymphoblastoid cell lines. Pull-down was directed against endogenously expressed PCBP1 and checked for the presence of endogenous HSPB1. IgG was used as a negative control. **b** Co-immunoprecipitation was performed on transiently transfected HeLa cell lines, clearly showing the binding of PCBP1 to HSPB1-WT and the increased binding to HSPB1-P182L. Pull down was directed against the V5-tag of the HSPB1 constructs and the presence of PCBP1 was checked with a VSV-tag. EGFP-V5 was used as a negative control. **c** Calculation of the PCBP1/HSPB1 ratio after quantification of the relative band intensities (*n* = 3). A One way ANOVA with Holm-Sidak multiple comparison test was performed to indicate the significant increased interaction of HSPB1-P182L with PCBP1. Data are represented as mean values with SD as error bars

To further investigate this novel HSPB1 interaction partner, immunoprecipitation (IP) was performed by the pull down of endogenous PCBP1 in patient-derived lymphoblastoid cell lines. Interestingly, a co-immunoprecipitation of the HSPB1-P182L mutant (in the C-terminal domain) and not for the HSPB1-R127W mutant (in the α-crystallin domain) and HSPB1 wild type (WT) was observed (Fig. 1a). This was further confirmed by the pull-down of HSPB1/EGFP, fused to a V5-tag in HeLa cell lines, transiently expressing HSPB1-WT, HSPB1-R127W, HSPB1-P182L and EGFP (a negative control). The HSPB1-R127W was chosen as an additional control for HSPB1-P182L, as this α-crystallin domain mutant is biological different and shows an increased chaperone activity and binding to client proteins, like tubulin, while HSPB1-P182L does not [2]. Interestingly, in HeLa cells the P182L mutant was more present in a complex with PCBP1, compared to HSPB1 wild type and HSPB1-R127W (Fig. 1b). These observations were further confirmed by western blot quantification (Fig. 1c). It still remains to be determined whether the interaction between HSPB1 and PCBP1 is direct or indirect.

When looking for co-expression of HSPB1 and PCBP1 in vivo, we found a stronger abundance of PCBP1 and HSPB1 during mouse embryonic development (Additional file 1: Figure S2), which further suggest that the interaction between these two proteins is taking place at specific moments in the cell, for example during the need for protein translation. Given the increased interaction of PCBP1 with the HSPB1-P182L mutant, we evaluated the effect of the P182L mutation on PCBP1 cellular localization, which is mostly present in the nucleus but also noticeable expressed in the cytosol. The PCBP1 localization is similar in fibroblasts of patients carrying the P182L mutation compared to control fibroblasts, without any apparent effect of the P182L mutation on the localization of HSPB1 and PCBP1 (Additional file 1: Figure S3). In addition, we assessed the expression of PCBP1 in differentiated neuroblastoma cell lines expressing either wild type or mutant HSPB1 (P182L or R127W). No differences were found in PCBP1 localization, nor had the mutations an effect on neurite outgrowth (Additional file 1: Figure S4). Notably, the formation of large protein aggregates, typically seen for C-terminal mutations in HSPB1 [1, 9], could not be detected in fibroblasts originating from the patient (HSPB1-P182L) or control individual, nor in the neuroblastoma cell lines stably expressing levels of the HSPB1 constructs that were comparable with the endogenous protein. We hypothesize that, because of its nature, this C-terminal mutation is more prone to from larger oligomeric complexes by the substitution of a polar with a hydrophobic amino acid. This makes it less solvent soluble and increases its interacting properties with other small heat shock proteins or other binding proteins. Depending on the cellular expression system used, the cell type and the presence of cellular stress, this mutation can lead more easily to protein aggregation.

### Mutant HSPB1 influences the biological activity of PCBP1

**Fig. 2:**
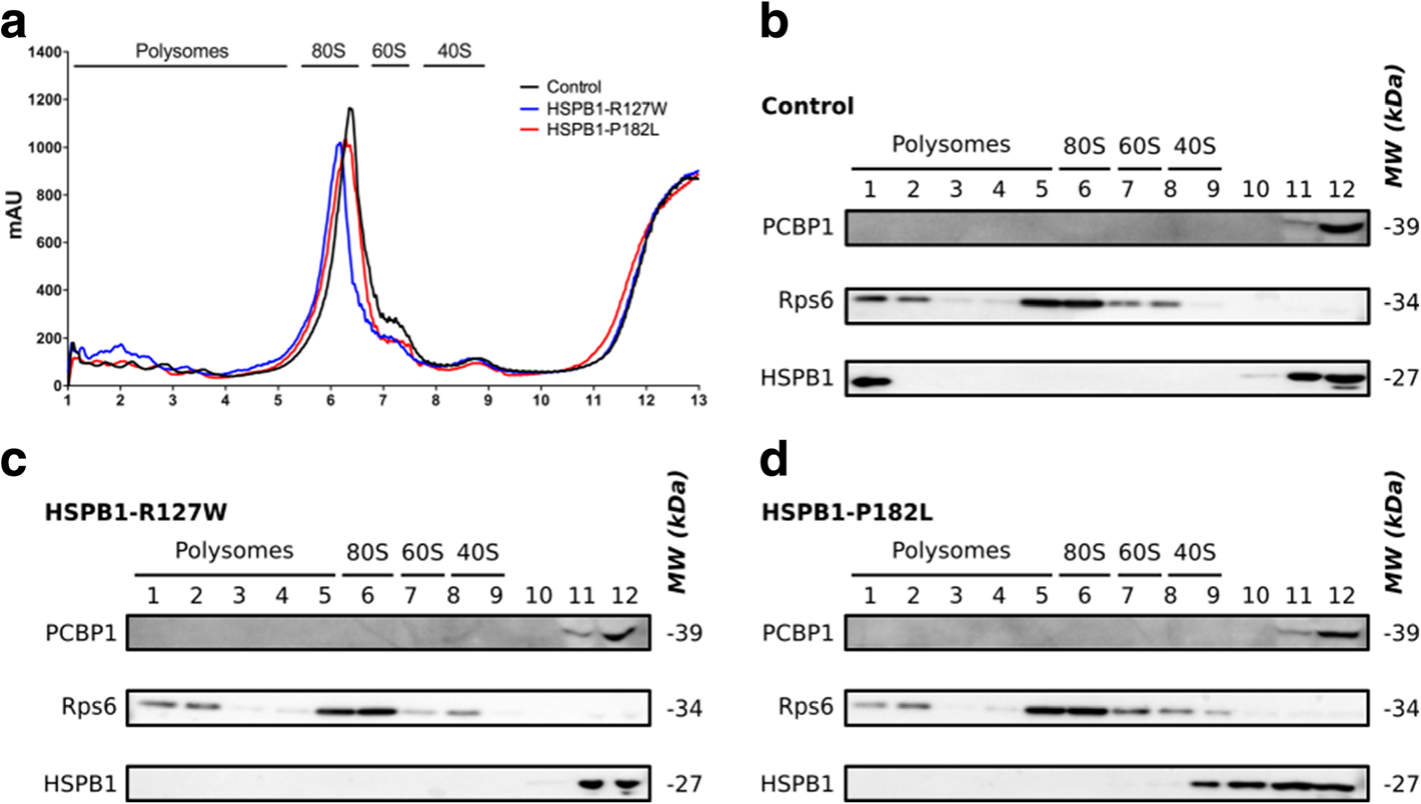
HSPB1 mutants do not show differences in general protein translation. **a** The polysome gradient analysis was performed on a 5-50% sucrose gradient with protein lysates originating from patient- and control-derived lymphoblastoid cell lines. **b**-**d** The isolated fractions originating from the polysome gradient analysis were assessed by western blotting. Rps6 was used as a ribosomal marker. The HSPB1 band visible in fraction 1 of the control individual (**b**) is due to technical variation

RNA binding proteins play a crucial role in the regulation of expression of many genes. One of the best-known functions of PCBP1 is its ability to repress translation by preventing the ribosomal units to dock and assemble on mRNA [16]. Therefore we investigated the effect of this increased interaction on total mRNA translation. We fractionated, by using sucrose density gradient centrifugation of patient-derived lymphoblastoid cells, the polysomes, monosomes, ribosomal subunits and ribonucleoproteins. A more active translation status of mRNAs would result in a more profound presence of ribosomes, and the Rps6 ribosomal marker, in the polysomal fractions. We saw no differences in total protein translation between the different genotypes when comparing mutant (HSPB1-R127W and HSPB1-P182L) to a healthy control individual (Fig. 2a). After loading the different isolated polysome fractions on gel, no differences in the distribution of PCBP1 could be demonstrated (Fig. 2b-d). PCBP1 was mostly present in the top fractions indicating that the protein is a monomer or part of small complexes, a pattern that was already reported before for PCBP1 [39] and other hnRNPs [25]. Interestingly, HSPB1-P182L shows a shift towards the heavier fractions containing the 40S ribosomal subunit, which might be explained by the nature of the mutation favoring complexes of larger oligomeric structures [9].

**Fig. 3:**
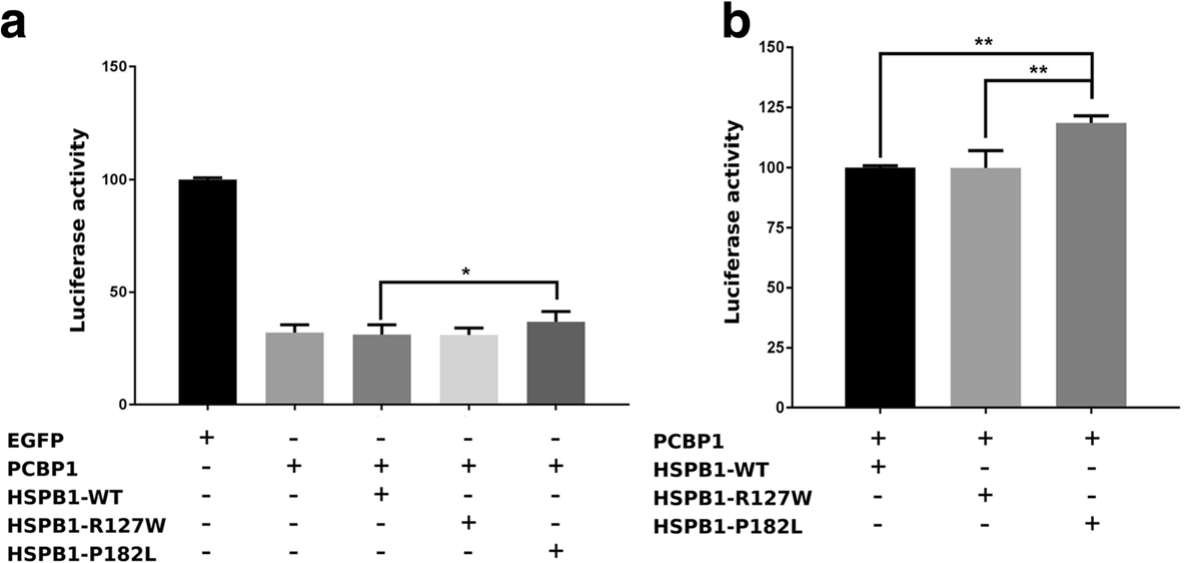
HSPB1-P182L reduces the translational repression activity of PCBP1. **a** Luciferase-based repression assay performed on HEK293 cell lines transiently transfected with PCBP1/EGFP alone or with PCBP1 together with wild type or mutant HSPB1. All data were subsequently normalized to the *renilla* luciferase and EGFP, which was used in this assay as a negative control. **b** Values from the same luciferase-based repression assay normalized to HSPB1-WT. All experiments were performed four times. Statistical analysis was performed by one way ANOVA with a Bonferroni multiple comparison test

To test for specific alterations in the activity of PCBP1, we performed a luciferase-based repression assay in HEK293 cells where mRNA recognition sequences for a PCBP1-MS2 fusion protein are cloned in the 3′UTR of the luciferase reporter gene. This approach enables us to study the effect of PCBP1 on the translation of the luciferase protein with or without the addition of wild type and mutant HSPB1. The expression of PCBP1 resulted in a severe reduction of the luciferase signal by approximately 68% when compared to an EGFP negative control (Fig. 3a). The conditions with and without HSPB1-WT or HSPB1-R127W, revealed no differences in the activity of PCBP1. Interestingly, a small but consistent loss in the activity of PCBP1 was observed when HSPB1-P182L was present (Fig. 3a). When comparing the luciferase values normalized to HSPB1-WT, it becomes clear that the control mutant HSPB1-R127W does not have an effect on the activity of PCBP1 (99,97 ± 5,09), but that HSPB1-P182L does and reduces the activity by approximately 20% (118,54 ± 2,97; Fig. 3b).

### Identification of PCBP1 target mRNAs from mouse brain

**Fig. 4:**
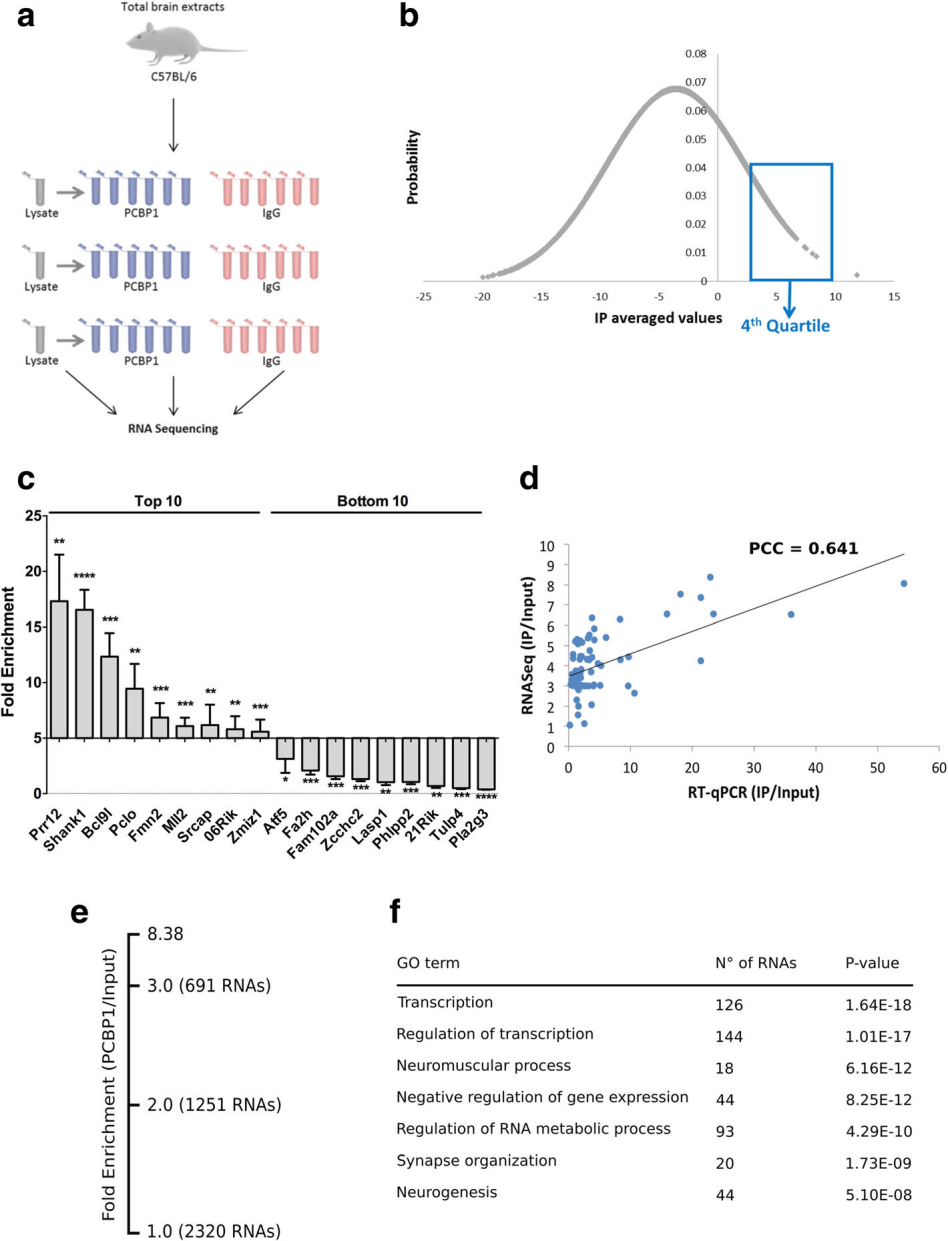
Identification and validation of PCBP1 mRNA targets in mouse brain. **a** Overview of the biochemical procedure to isolate endogenous PCBP1 and identify its mRNA targets. Normal goat IgG was taken along as a negative control. RNA immunoprecipitation experiments were performed *in triplo*, with three different C57Bl/6 mouse brains, in order to rule out biological variation. **b** Data originating from RNA sequencing were normalized to the expression level of genes identified in the total brain lysates and to the negative control, IgG. The 4th quartile (top 25%) of the identified genes was selected for the validation of the performed data analysis. **c** Ten top and bottom genes of the 4th quartile selected from the total pool of identified mRNA targets were validated by RNA immunoprecipitation and RT-qPCR, showing the same trend in expression levels as seen in the RIP sequencing data. Data were normalized by subtracting the input values from the IP and IgG values. Afterwards these values were corrected for the amplification efficiencies (AE) of the selected genes; AE^[−(IP-input)] and AE^[−(IgG-input)]. Normalized and corrected IP values are shown in the graph. Multiple *t*-test with Holm-Sidak as a correction for multiple comparisons was used to test for the enrichment targets in the IP versus IgG. **d** Correlation of identified mRNA targets obtained by RNA sequencing versus RT-qPCR. Data values originating from RIP sequencing and RIP followed by RT-qPCR were normalized to their input values (IP/Input). Each point represents an individual mRNA, which was quantified using both methods. PCC = Pearson’s correlation coefficient. **e** Overview of the number of identified mRNA targets ≥ 2-fold (PCBP1/Input). Note that the fold enrichment values in the graph are presented in log2 scale while the values discussed in the text are in linear scale. **f** Selected GO term enrichments observed for PCBP1-associated mRNAs. RNAs enriched ≥ 2-fold (PCBP1/Input) were used

To try to understand the effect of HSPB1-P182L on the mRNA targets regulated by PCBP1, we aimed to identify its binding transcripts. For that, we immunoprecipitated PCBP1 from adult mouse brain and isolated the bound mRNA. Total mouse brain was used instead of sciatic nerve or spinal cord because of too low starting material in the latter two tissues. In three independent experiments, total RNA was isolated from the IP and analyzed by RNA sequencing (RIP sequencing). Pre-immune serum coupled beads (IgG) was used as a negative control to account for the background mRNAs (Fig. 4a). To validate our results we confirmed by RT-qPCR the expression profiles of the top 10 and bottom 10 genes, present in the 4th quartile of the list containing transcripts identified by RIP sequencing (Fig. 4b-c). The correlation between RT-qPCR and RNA sequencing data for 50 additional genes (Additional file 2: Table S1) was highly significant (Pearson’s correlation coefficient = 0,641; Fig. 4d), indicating that the RNA sequencing data are robust and reliable. The identified mRNAs were ranked by enrichment in the IP relative to input and normalized to the negative control IgG (Additional file 3: Table S2). This allowed us to identify a total of 2,320 mRNAs significantly enriched in the PCBP1 IP (using an average of ≥ 2-fold enrichment as a cutoff across three IPs and an adjusted p-value ≤ 0,05; Fig. 4e). This set represented approximately 7,4% of mRNAs expressed in the input fractions.

Although RBPs are forming dynamic protein complexes, increasing evidence demonstrate that individual RBPs can regulate a biologically coherent set of target mRNAs in order to coordinate their expression profiles [19, 26]. We therefore performed DAVID Gene Ontology (GO) term analysis on the targets of PCBP1 identified by RIP sequencing (≥2-fold). A significant enrichment of several GO term categories was found, including transcription and regulation of transcription (p-values of 1,64 × 10^−18^ and 1,01 × 10^−17^, respectively; Fig. 4f). Interestingly, several GO term categories were associated with neuronal processes like neuromuscular process, synapse organization and neurogenesis. RT-qPCR analysis confirmed the genes belonging to these selected GO term categories as being significant PCBP1 targets. Interestingly, there was a strong association of Shank1 and Shank3 (*p* ≤ 0,0001 and *p* ≤ 0,01 respectively) with PCBP1 (Additional file 1: Figure S5). These proteins are present in the synapse and crucial in making the connection between the actin cytoskeleton and neurotransmitter receptors [33]. In addition, *Shank1* null mutant mice display reduced motor coordination and neuromuscular strength [46]. Taking together there is a strong indication that PCBP1 plays a role in regulating the expression profile of several genes in the synapses of neuronal cells.

### PCBP1 mRNA targets have larger UTRs and share a common recognition sequence

**Fig. 5:**
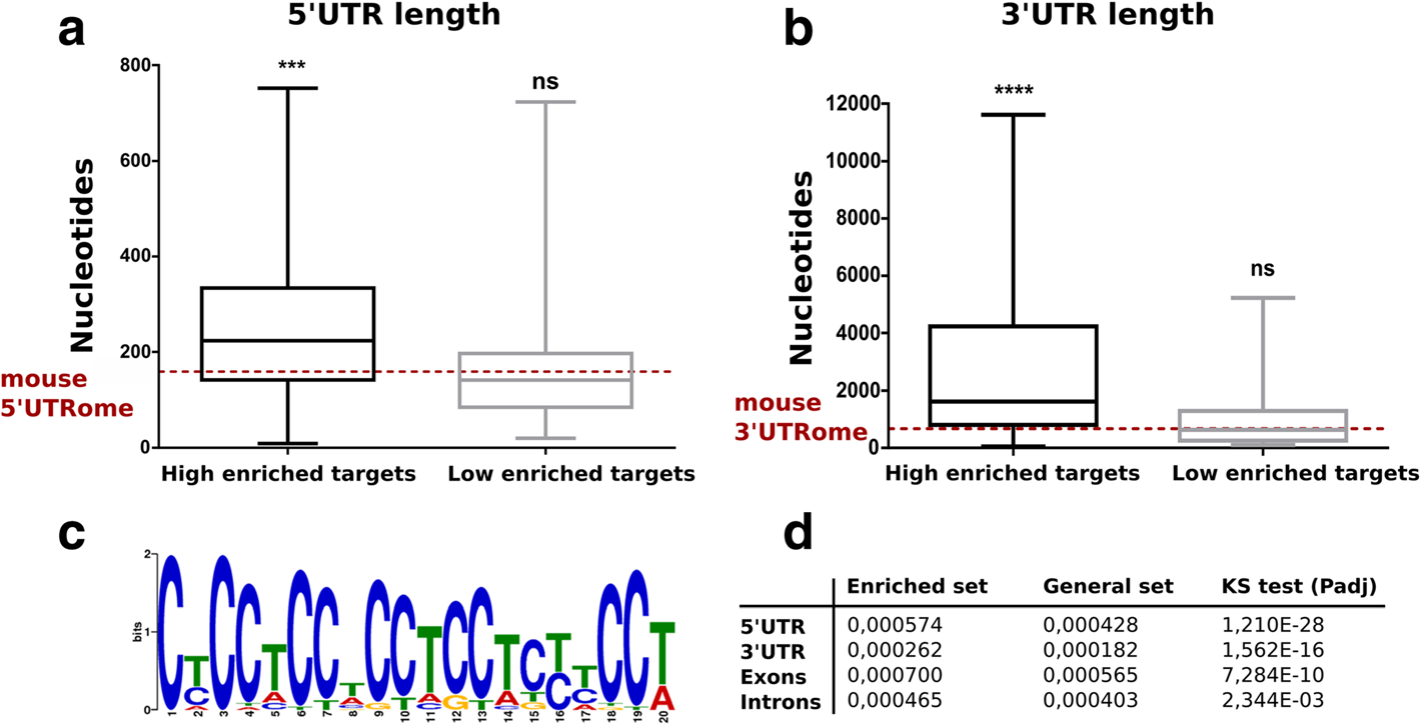
PCBP1 mRNA targets have larger UTRs and share a common recognition sequence. **a** The average length of the 5′UTRs present in the top 50 high and low enriched genes was compared to the average length of the mouse 5′UTRome. Wilcoxon rank sum test, *p* ≤ 0,001. The same analysis was performed in (**b**) with the 3′UTR present in the top 50 genes of high and low enriched genes. Wilcoxon rank sum test; *p* ≤ 0,001. **c** PCBP1 mRNA targets share a common recognition sequence that is rich in poly(C) repeat stretches. This RNA motif was identified by subjecting the most stringent set of PCBP1 targets, above ≥ 2-fold, to MEME analysis. **d** Table with statistics showing the enrichment of the RNA motif in values

In order to investigate how PCBP1 might regulate specific transcripts in the neuron, we searched for structural elements enriched in PCBP1 mRNA targets. RNA binding proteins have a preference to bind to the 5′- and 3′-UTR of transcripts. Depending on the UTR where it binds, it can have multiple different functions [10]. Here, we took advantage of our three independent RNA sequencing experiments to select the most stringent set of targets. We selected those mRNAs that were highly enriched in the PCBP1 IP but in addition we selected as well the targets that were less enriched. This resulted in 103 targets that were subjected to a database search (http://utrdb.ba.itb.cnr.it; Additional file 4: Table S3). Interestingly, and in line with what was recently reported for *Staufen* targets [18, 30], the average length of the 5′-UTR of PCBP1 mRNA targets was significantly larger than that in the mouse 5′UTRome (258 bases for PCBP1 targets versus 160 bases for the mouse 5′UTRome, Wilcoxon rank sum test *p* ≤ 0,0001; Fig. 5a). In addition, our data showed an enrichment of mRNA targets bound by PCBP1 with long 3′UTRs when compared to the average length of the mouse 3′-UTR (2,881 bases for PCBP1 targets versus 607 bases for the mouse 3′UTRome, Wilcoxon rank sum test *p* ≤ 0,001; Fig. 5b). This observation was not seen for the less enriched transcripts, suggesting that PCBP1 specifically binds to transcripts that have larger UTRs.

We next investigated whether PCBP1 preferentially binds to specific regions in the larger appreciated UTRs. We therefore selected the most stringent set of targets, which was used for the MEME prediction software (http://meme-suite.org). We found that PCBP1 targets were highly enriched with a specific poly(C) stretch (CTCCTCCTCCTCC), pointing to the presence of an RBP binding sequence (Fig. 5c). The location of the poly(C) stretch was spread among the entire sequence of the analysed targets but with a strong enrichment in the 3′- and 5′-UTR. Motif frequency calculations were performed on the sequences extracted (for exons, 5′UTRs, 3′UTRs, introns) from Biomart and PerlAPI (Ensembl). This was done for two group of genes, representing the ‘enriched set’ and the ‘general set’. From the total of 1,252 differentially expressed genes (≥2-fold enrichment), 1,060 genes (4,524 transcripts) had complete data and the motif frequencies in exons, 5′UTR, 3′UTR and introns were calculated (Fig. 5d; table ‘Enriched set’). The second set contained all the remaining 51,224 transcripts in 16,381 genes (Fig. 5d; table ‘General set’). We generated 100 random sets of 4,524 genes from the ‘General set’. The distribution of each random set was compared with the distribution of the ‘Enriched set’ set using signed two-sample Kolmogorov-­Smirnov test, for exons, 5′UTRs, 3′UTRs and introns, respectively. The frequencies of the motif in the ‘Enriched set’ were systematically greater than in the random sets for all features, with the exception of 3 comparisons in the motif frequencies in 5’UTRs, where the Benjamini-Hochberg corrected p­value did not reach significance level of 0,05. The enrichment of the motif in the differentially expressed transcripts for all features was unequivocally supported by the statistical test (average of adjusted p­value for 100 comparisons; in exons < 7,41 × 10^−10^, in 5′UTR < 1,21 × 10^−28^, in 3′UTR < 1,64 × 10^−16^ and in introns < 0,003; Fig. 5d). These data indicate that the identified motif of PCBP1 is mainly present in the 5′ and 3′UTR of its target genes and therefore supports for an important role in translational control and mRNA stabilisation.

### PCBP1 targets known neuropathy genes

**Fig. 6:**
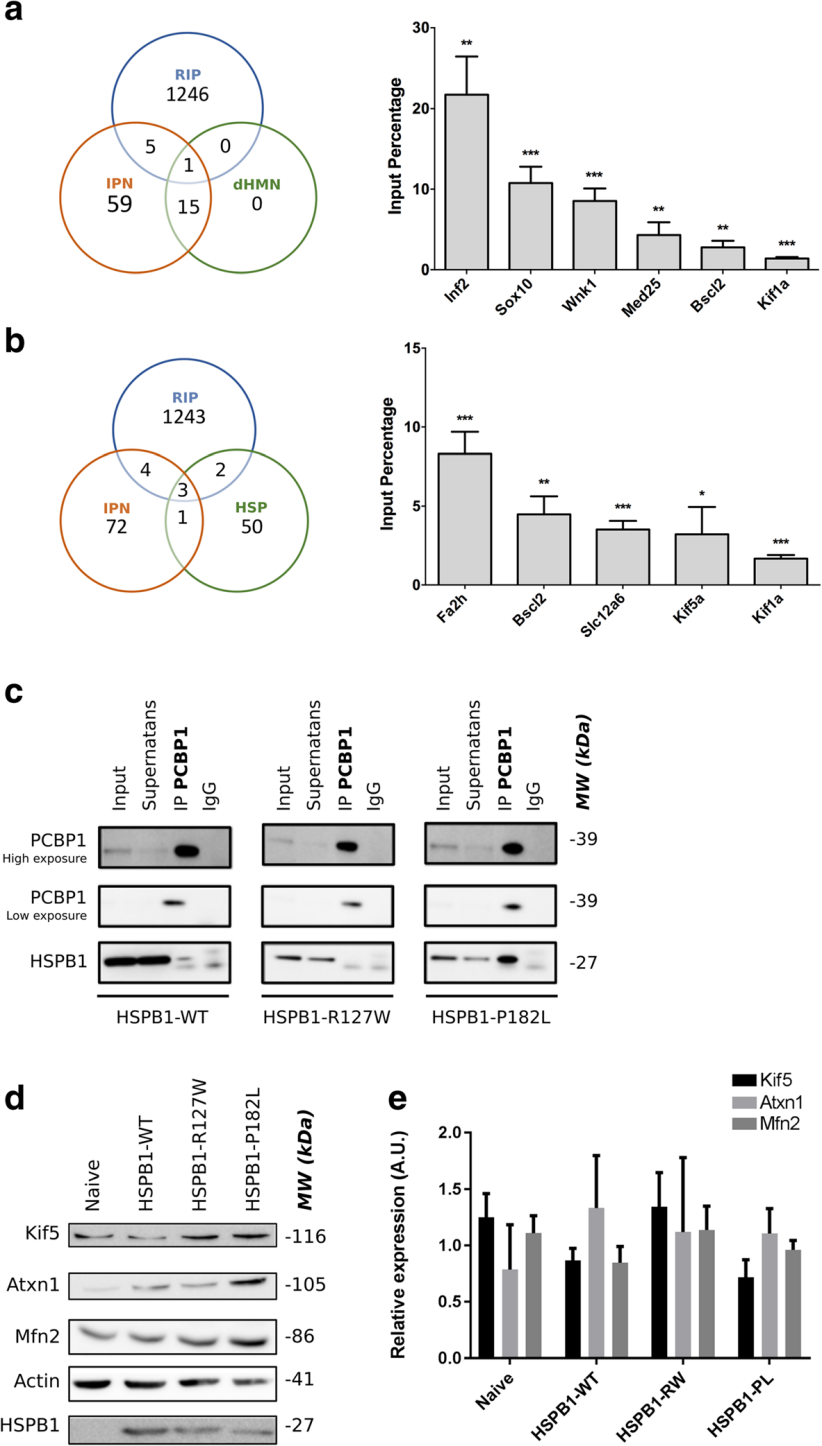
PCBP1 controls the expression of known neuropathy genes. **a** Venn diagram showing the overlap between genes identified through RIP sequencing (≥2 fold) and known IPN and dHMN genes. Overlapping genes were confirmed by RNA immunoprecipitation of PCBP1 followed by RT-qPCR on total mouse brain. Raw Ct values originating from mRNA targets when PCBP1 was pulled down were corrected for the pull down of IgG and normalized for the Ct values in the total lysate used for the pull down experiment. These obtained values are shown as input percentage values. **b** The same approach was done for an additional dataset with all known genes causing HSP. **c** Co-immunoprecipitation was performed on NSC-34 cells stably transduced with HSPB1 wild type and mutants. Pull-down was directed against endogenous PCBP1 and the presence of exogenous HSPB1 was investigated. **d** The expression level of a selection of identified PCBP1 targets was checked in stably transduced NSC-34 cell lines by western blot and by RT-qPCR (**e**). Values are shown as mean with SD as error bar. Multiple *t*-test with Holm-Sidak as a correction for multiple comparisons was used as statistical test

To date more than 80 disease-associated genes have been identified for Inherited Peripheral Neuropathies (IPN) including CMT and closely related diseases [49]. The constant discovery of novel genes and mutations being the cause of this neurological disease suggests a pleiotropic repertoire of proteins being affected and causing a similar phenotype. Therefore we looked at the presence of these known disease-associated genes in our dataset of genes identified through RNA sequencing (≥2 fold). Interestingly, we saw an overlap between the two datasets for six genes (*Inf2, Sox10, Wnk1, Med25, Kif1a* and *Bscl2)*, where the latter one was specifically causing a pure dHMN phenotype. These six mRNAs were validated by RT-qPCR after RNA immunoprecipitation of PCBP1 on adult mouse brain samples, and were found to be significantly associated with PCBP1 (Fig. 6a).

Hereditary Spastic Paraplegias (HSPs) are clinically and genetically heterogeneous disorders with a progressive weakness and spasticity of the lower limbs. Similar to CMT2/dHMN, the major neuropathological feature of HSP is axonal degeneration that is maximal in the terminal portions of the longest descending and ascending neurons [28, 48]. Therefore, we also aimed looking for the presence of genes known to be associated with HSP in our dataset (≥2 fold). This search resulted in the identification of five overlapping genes (*Fa2h, Slc12a6, Kif5a, Kif1a and Bscl2*), where the latter two were previously reported as CMT2/dHMN causing genes [42, 52]. All the five mentioned genes were detected significantly by RT-qPCR (Fig. 6b).

To further study the consequences of the increased interaction between HSPB1-P182L and PCBP1, we investigated the protein expression level of some PCBP1 targets in mouse NSC-34 motorneuron-like cell lines expressing HSPB1 wild type and mutant proteins. We confirmed the increased interaction, seen in patient-derived lymphoblasts, by performing a co-immunoprecipitation for PCBP1 on stable NSC-34 cell lines (Fig. 6c). Because of antibody restrictions we were limited to Mfn2, Atxn1 and Kif5 to investigate their protein expression levels by western blot. These three genes were identified as PCBP1 mRNA targets and validated by RIP-qPCR (data not shown). Interestingly, an increased protein expression for these genes was observed in cells carrying the P182L mutation (Fig. 6d), which was not seen at the mRNA level (Fig. 6e). This increased protein expression might be a direct consequence of the increased interaction between the P182L mutant and PCBP1 leading to a loss of translational repression.

## Discussion

This study reports the poly(C)binding protein 1 (PCBP1) as a novel binding partner for HSPB1, which forms an increased interaction with mutant HSPB1-P182L causing dHMN. We identified this novel interaction by performing a tandem affinity purification experiment coupled to mass spectrometry. The availability of patient-derived material enabled us to confirm our findings and to further study the biochemical consequences of this interaction. In basal conditions we could not detect any differences in global protein translation, nor was there a difference seen in the distribution of HSPB1 and PCBP1 in the different fractions after the separation of polysomes and ribosomal subunits by sucrose gradient centrifugation. These data suggest that the identified interaction between mutant P182L and PCBP1 leads to subtle effects that are not noticeable in white blood cells having a lower complexity than neurons. A more specific approach was chosen to test for the direct consequences on the function of PCBP1. A luciferase-based transcriptional repression assay showed that PCBP1 gives approximately 20% less translational repression when HSPB1-P182L is present. In order to investigate the possible outcomes of this effect on the mRNA targets of PCBP1, RNA immunoprecipitation coupled to RNA sequencing was performed. Total mouse brain was used because of the limited amount of starting material in sciatic nerve and spinal cord. In total 2,320 mRNA targets (above 2-fold) were identified, all showing a significant larger 5′ and 3′UTR when compared to the average UTR length. Such an enrichment for larger UTRs has also been reported for Stau2 mRNA targets, which is an RNA binding protein directing local protein translation at neuronal synapses. These larger UTRs were enriched for primary and secondary structures important for the binding of Stau2 [18]. Indeed, an RNA recognition motif (CTCCTCCTCCTCC) was highly enriched in the 5′ and 3′UTR of PCBP1 mRNA targets, but to a lesser extent in exons and introns. Defining a conclusive RNA recognition motif for PCBP1 has been found difficult as many reports have identified different kinds of motifs [51, 55]. Interestingly, all the reported motifs for PCBP1 are highly enriched for cytosine. It has been shown before that RNA structures composed out of polycytosines are more prone to form hairpin loops [35]. Most likely there is a commonality in the secondary structures formed by the multiple identified PCBP1 recognition motifs. It is therefore suggestive that the recognition of mRNA by PCBP1, and RNA binding proteins in general, is largely mediated by the secondary structure of the mRNA rather than its primary structure. Besides the clear presence of neuronal transcripts seen among the mRNA targets containing PCBP1 recognition motifs, we identified known genes associated with hereditary peripheral neuropathies and hereditary spastic paraplegias. Although our approach to identify PCBP1 mRNA targets started from mouse brain and not from peripheral nerve tissue material, we were able to identify genes that showed an increased protein expression level in mouse motor neuronal cells carrying the HSPB1-P182L mutation. This change in protein expression hints to a direct consequence of the increased interaction between mutant HSPB1 and PCBP1. Our approach suggests that whole brain tissue material can be used for explorative experiments, like RIP sequencing, when studying RNA binding proteins involved in motor neuron diseases. Similar results where obtained for TDP-43 mRNA targets that were identified using cortical neurons that showed an altered expression profile in motor neurons when TDP-43 was mutated [45].

The regulation of PCBP1 has recently been found to be of crucial importance in turning solid tumor cells into metastatic cells. Altering the expression profile of specific mRNA targets inhibits the process of metastasis [22, 23, 47]. Similar to the control of mRNAs responsible for the transition of cancer cells, it has been shown that incorrect processing, delivery and regulation of mRNAs can be the cause of human neurological diseases [53]. Looking at the strong potential of PCBP1 to control the fate of many mRNAs, it is not surprising that it also controls transcripts important for neuronal processes and consequently known neuropathy genes. This study identified a set of ubiquitously expressed genes that are controlled by PCBP1 and contain diverse neuron specific functions, going from axonal transport to the assistance of neuronal myelin formation. Conditional knock-out studies in mice of some of the identified genes (*Kif1a, Kif5a* and *Slc12a6*) all lead to the development of neurodegeneration, often associated with the loss of motor and sensory neurons [12, 54, 56]. Not all identified genes lead to neuropathy phenotypes caused by a reduced expression or loss of protein function. More specifically, mutations reported in *Bscl2* clearly affect N-glycosylation of the protein favoring more its wild type function by increasing its localization in the endoplasmatic reticulum [52]. Mutations found in *Inf2* can result both in a loss or gain of function as the net result of the mutations are an increase in filamentous actin. This increase can be caused by an increase in polymerization activity or a decrease in the depolymerization activity of mutant Inf2 [34]. Interestingly, some of the identified targets (*Kif5, Atxn1* and *Mfn2)* of PCBP1 clearly show a loss of translational repression in motorneuron-like cells carrying the HSPB1-P182L mutation, as shown by their increase in protein expression. This indicates that the effect of the HSPB1 mutation on the targets of PCBP1 has direct consequences on motorneuron-like cells. Nevertheless, precautions have to be made when linking PCBP1 to the expression levels of the identified neuropathy genes. Besides its strong role in translational control, which could be enhancing or repressive, the role of PCBP1 in the transport and stability of mRNA has to be evaluated as well.

It remains to be determined how the C-terminal P182L mutation in HSPB1 can affect the binding strength to other proteins. So far no studies that detail the molecular structure of human HSPB1 were reported, and thus the function of its C-terminal extension remains unclear. In contrast to the N-terminal and the α-crystallin domain, the C-terminal extension of HSPB1 displays a large variability in length, sequence conservation and structure [9]. Despite the low conservation among species, the C-terminal extension contains a well-conserved IXI/V motif that interacts with regions located on neighboring monomers [38]. It is most likely that this motif mediates for a large part the crucial binding properties of the C-terminal extension to other monomers when forming oligomeric structures and to other interacting proteins. The P182L/S mutations reported so far [14, 27] are located in the middle of this conserved IXI/V motif and lead to a dramatic changes in the oligomeric structure of HSPB1 [9]. The T180I and R188W are two other mutations that are located in the C-terminal extension of HSPB1 [6, 32]. Interestingly, the T180I mutation is located on the border of the conserved tripeptide IXI/V motif and consequently leads to the creation of a group of three consecutive isoleucine residues. Similarly, the R188W mutation leads to a structural change of the C-terminal extension by introducing a bulky aromatic amino acid. Because of the extreme flexible and unstructured nature of the C-terminal extension it is possible that altering the organization of amino acids creates disturbances in the protein binding capabilities of this molecular chaperone. Besides a prominent need for flexibility and structure variability, the C-terminal extension of HSPB1 is also characterized by a high degree of polar residues. Interestingly, this extension is typically pointing to the outside when oligomers are being created [8]. By doing so, it counteracts the hydrophobicity of the created oligomer and keeps it soluble by interacting with the polar environment [36]. Therefore, it is of utmost importance that the C-terminal extension remains polar to maintain environmental interactions. Interestingly, it has been shown in *Xenopus laevis* that the chaperone activity of Hsp30C is drastically impaired when the polarity of its C-terminal extensions is reduced [15]. This highlights the importance of the polar residues in the C-terminus of sHSPs and probably also of HSPB1. Interestingly, all reported mutations in the C-terminal extension of HSPB1 (T180I, P182L/S and R188W) results in the drastic change from a polar, or positively charged amino acid, to a hydrophobic amino acid. It is suggestive that this reduction in polarity causes the HSPB1 oligomer to make less contact with solvents in its environment, making it less soluble. This reduced solubility can consequently increase the affinity for other binding proteins. The decrease in solubility of HSPB1-P182L, together with an alteration of the conserved IXI/V motif probably creates a mutant protein that affects its binding strength to other proteins, such as our identified increased interaction with PCBP1.

## Conclusions

We report a novel interaction between mutant HSPB1-P182L and the RNA binding protein PCBP1, leading to a reduction in its translational repression activity. Identifying PCBP1 mRNA targets revealed a marked prevalence for an RNA recognition motif, preferably seen in their 5′ and 3′UTRs. Among many neuronal transcripts we identified known genes associated with hereditary peripheral neuropathies and hereditary spastic paraplegias. We hypothesize that mutant HSPB1 can mediate translational repression by forming an interaction with PCBP1 leading to an alteration in the expression levels of genes important to cause peripheral neuropathies. These findings further support a role for mutant HSPB1 in neurodegenerative diseases.

## Additional files

Additional file 1: Figure S1. HSPB1 and PCBP1 are ubiquitously expressed proteins. **Figure S2**: The expression of PCBP1 and HSPB1 increases during embryonal development. **Figure S3**: Patient fibroblasts show no protein aggregation. **Figure S4**: PCBP1 shows no abnormal localization in stable neuroblastoma cell lines. **Figure S5**: PCBP1 mRNA targets are enriched in neuronal processes. (DOCX 2584 kb)
Additional file 2: Table S1. Full list of PCBP1 target mRNAs validated by RT-qPCR for enrichment in the PCBP1 IP (Relates to Fig. 4). (DOC 52 kb)
Additional file 3: Table S2. mRNAs enriched more than 2-fold in the PCBP1 IP relative to input. Relates to Fig. 4. (XLSX 74 kb)
Additional file 4: Table S3. PCBP1 mRNA targets used for mouse UTR database search. Relates to Fig. 5. (DOC 80 kb)

## References

[1] Ackerley S, James PA, Kalli A, French S, Davies KE, Talbot K (2006). A mutation in the small heat-shock protein HSPB1 leading to distal hereditary motor neuronopathy disrupts neurofilament assembly and the axonal transport of specific cellular cargoes. Hum Mol Genet.

[2] Almeida-Souza L, Asselbergh B, d’Ydewalle C, Moonens K, Goethals S, De Winter V (2011). Small heat-shock protein HSPB1 mutants stabilize microtubules in Charcot-Marie-Tooth neuropathy. J Neurosci.

[3] Almeida-Souza L, Asselbergh B, De Winter V, Goethals S, Timmerman V, Janssens S (2013). HSPB1 facilitates the formation of non-centrosomal microtubules. PLoS One.

[4] Almeida-Souza L, Goethals S, De Winter V, Dierick I, Gallardo R, Van Durme J (2010). Increased monomerization of mutant HSPB1 leads to protein hyperactivity in Charcot-Marie-Tooth neuropathy. J Biol Chem.

[5] Arrigo AP, Virot S, Chaufour S, Firdaus W, Kretz-Remy C, Diaz-Latoud C Hsp27 consolidates intracellular redox homeostasis by upholding glutathione in its reduced form and by decreasing iron intracellular levels. Antioxid Redox Signal. 7:414–22. doi:10.1089/ars.2005.7.414 10.1089/ars.2005.7.414 15706088

[6] Capponi S, Geroldi A, Fossa P, Grandis M, Ciotti P, Gulli R (2011). HSPB1 and HSPB8 in inherited neuropathies: study of an Italian cohort of dHMN and CMT2 patients. J Peripher Nerv Syst.

[7] Capponi S, Geuens T, Geroldi A, Origone P, Verdiani S, Cichero E (2016). Molecular Chaperones in the Pathogenesis of Amyotrophic Lateral Sclerosis: the role of HSPB1. Hum Mutat.

[8] Carver JA, Aquilina JA, Truscott RJ, Ralston GB (1992). Identification by 1H NMR spectroscopy of flexible C-terminal extensions in bovine lens alpha-crystallin. FEBS Lett.

[9] Chalova AS, Sudnitsyna MV, Strelkov SV, Gusev NB (2014). Characterization of human small heat shock protein HspB1 that carries C-terminal domain mutations associated with hereditary motor neuron diseases. Biochim Biophys Acta.

[10] Chaudhury A, Hussey GS, Ray PS, Jin G, Fox PL, Howe PH (2010). TGF-beta-mediated phosphorylation of hnRNP E1 induces EMT via transcript-selective translational induction of Dab2 and ILEI. Nat Cell Biol.

[11] Dierick I, Baets J, Irobi J, Jacobs A, De Vriendt E, Deconinck T (2008). Relative contribution of mutations in genes for autosomal dominant distal hereditary motor neuropathies: a genotype-phenotype correlation study. Brain.

[12] Ding J, Delpire E (2014) Deletion of KCC3 in parvalbumin neurons leads to locomotor deficit in a conditional mouse model of peripheral neuropathy associated with agenesis of the corpus callosum. BehavBrainRes. 128–36. doi:10.1016/j.bbr.2014.08.005 10.1016/j.bbr.2014.08.005 PMC4179972 25116249

[13] Edvardson S, Hama H, Shaag A, Gomori JM, Berger I, Soffer D (2008). Mutations in the fatty acid 2-hydroxylase gene are associated with leukodystrophy with spastic paraparesis and dystonia. Am J Hum Genet.

[14] Evgrafov OV, Mersiyanova I, Irobi J, Van Den Bosch L, Dierick I, Leung CL (2004). Mutant small heat-shock protein 27 causes axonal Charcot-Marie-Tooth disease and distal hereditary motor neuropathy. Nat Genet.

[15] Fernando P, Abdulle R, Mohindra A, Guillemette JG, Heikkila JJ (2002). Mutation or deletion of the C-terminal tail affects the function and structure of Xenopus laevis small heat shock protein, hsp30. Comp Biochem Physiol B Biochem Mol Biol.

[16] Geuens T, Bouhy D, Timmerman V (2016). The hnRNP family: insights into their role in health and disease. Hum Genet.

[17] Haslbeck M, Franzmann T, Weinfurtner D, Buchner J (2005). Some like it hot: the structure and function of small heat-shock proteins. Nat Struct & 38. Mol Biol.

[18] Heraud-Farlow JE, Sharangdhar T, Li X, Pfeifer P, Tauber S, Orozco D (2013). Staufen2 regulates neuronal target RNAs. Cell Rep.

[19] Hogan DJ, Riordan DP, Gerber AP, Herschlag D, Brown PO (2008). Diverse RNA-binding proteins interact with functionally related sets of RNAs, suggesting an extensive regulatory system. PLoS Biol.

[20] Holmgren A, Bouhy D, De Winter V, Asselbergh B, Timmermans J-P, Irobi J (2013). Charcot-Marie-Tooth causing HSPB1 mutations increase Cdk5-mediated phosphorylation of neurofilaments. Acta Neuropathol.

[21] Howard HC, Mount DB, Rochefort D, Byun N, Dupré N, Lu J (2002). The K-Cl cotransporter KCC3 is mutant in a severe peripheral neuropathy associated with agenesis of the corpus callosum. Nat Genet.

[22] Howley BV, Hussey GS, Link LA, Howe PH (2016). Translational regulation of inhibin βA by TGFβ via the RNA-binding protein hnRNP E1 enhances the invasiveness of epithelial-to-mesenchymal transitioned cells. Oncogene.

[23] Hussey GS, Chaudhury A, Dawson AE, Lindner DJ, Knudsen CR, Wilce MCJ (2011). Identification of an mRNP complex regulating tumorigenesis at the translational elongation step. Mol Cell.

[24] Irobi J, Dierick I, Jordanova A, Claeys KG, De Jonghe P, Timmerman V (2006). Unraveling the genetics of distal hereditary motor neuronopathies. Neuromolecular Med.

[25] Katahira J, Miki T, Takano K, Maruhashi M, Uchikawa M, Tachibana T (2008). Nuclear RNA export factor 7 is localized in processing bodies and neuronal RNA granules through interactions with shuttling hnRNPs. Nucleic Acids Res.

[26] Keene JD (2007). RNA regulons: coordination of post-transcriptional events. Nat Rev Genet.

[27] Kijima K, Numakura C, Goto T, Takahashi T, Otagiri T, Umetsu K (2005). Small heat shock protein 27 mutation in a Japanese patient with distal hereditary motor neuropathy. J Hum Genet.

[28] Klebe S, Stevanin G, Depienne C Clinical and genetic heterogeneity in hereditary spastic paraplegias: from SPG1 to SPG72 and still counting. Rev Neurol (Paris). 171:505–30. doi:10.1016/j.neurol.2015.02.017 10.1016/j.neurol.2015.02.017 26008818

[29] Lafreniere RG, MacDonald MLE, Dube M-P, MacFarlane J, O’Driscoll M, Brais B (2004). Identification of a novel gene (HSN2) causing hereditary sensory and autonomic neuropathy type II through the study of canadian genetic isolates. Am J Hum Genet.

[30] Laver JD, Li X, Ancevicius K, Westwood JT, Smibert CA, Morris QD, Lipshitz HD (2013). Genome-wide analysis of Staufen-associated mRNAs identifies secondary structures that confer target specificity. Nucleic Acids Res.

[31] Leal A, Huehne K, Bauer F, Sticht H, Berger P, Suter U (2009). Identification of the variant Ala335Val of MED25 as responsible for CMT2B2: molecular data, functional studies of the SH3 recognition motif and correlation between wild-type MED25 and PMP22 RNA levels in CMT1A animal models. Neurogenetics.

[32] Luigetti M, Fabrizi GM, Madia F, Ferrarini M, Conte A, Del Grande A (2010). A novel HSPB1 mutation in an Italian patient with CMT2/dHMN phenotype. J Neurol Sci.

[33] MacGillavry HD, Kerr JM, Kassner J, Frost NA, Blanpied TA (2016). Shank-cortactin interactions control actin dynamics to maintain flexibility of neuronal spines and synapses. Eur J Neurosci.

[34] Mathis S, Funalot B, Boyer O, Lacroix C, Marcorelles P, Magy L (2014). Neuropathologic characterization of INF2-related Charcot-Marie-Tooth disease: evidence for a Schwann cell actinopathy. J Neuropathol Exp Neurol.

[35] Melnykov AV, Nayak RK, Hall KB, Van Orden A (2015). Effect of loop composition on the stability and folding kinetics of RNA hairpins with large loops. Biochemistry.

[36] Morris AM, Treweek TM, Aquilina JA, Carver JA, Walker MJ (2008). Glutamic acid residues in the C-terminal extension of small heat shock protein 25 are critical for structural and functional integrity. FEBS J.

[37] Nefedova VV, Muranova LK, Sudnitsyna MV, Ryzhavskaya AS, Gusev NB (2015). Small heat shock proteins and distal hereditary neuropathies. Biochemistry (Mosc).

[38] Pasta SY, Raman B, Ramakrishna T, Rao CM (2004). The IXI/V motif in the C-terminal extension of alpha-crystallins: alternative interactions and oligomeric assemblies. Mol Vis.

[39] Perlewitz A, Nafz B, Skalweit A, Fähling M, Persson PB, Thiele B-J (2010). Aldosterone and vasopressin affect {alpha}- and {gamma}-ENaC mRNA translation. Nucleic Acids Res.

[40] Pingault V, Bondurand N, Kuhlbrodt K, Goerich DE, Préhu MO, Puliti A (1998). SOX10 mutations in patients with Waardenburg-Hirschsprung disease. Nat Genet.

[41] Reid E, Kloos M, Ashley-Koch A, Hughes L, Bevan S, Svenson IK (2002). A kinesin heavy chain (KIF5A) mutation in hereditary spastic paraplegia (SPG10). Am J Hum Genet.

[42] Rivière J-B, Ramalingam S, Lavastre V, Shekarabi M, Holbert S (2011). KIF1A, an axonal transporter of synaptic vesicles, is mutated in hereditary sensory and autonomic neuropathy type 2. Am J Hum Genet.

[43] Salmon P, Trono D (2006) Production and titration of lentiviral vectors. Curr Protoc Neurosci. Chapter 4:Unit 4.21. doi:10.1002/0471142301.ns0421s37. 10.1002/0471142301.ns0421s37 18428637

[44] Schneider CA, Rasband WS, Eliceiri KW (2012). NIH Image to ImageJ: 25 years of image analysis. Nat Methods.

[45] Sephton CF, Cenik C, Kucukural A, Dammer EB, Cenik B, Han Y (2011). Identification of neuronal RNA Targets of TDP-43-containing ribonucleoprotein complexes. J Biol Chem.

[46] Silverman JL, Turner SM, Barkan CL, Tolu SS, Saxena R, Hung AY, Sheng M, Crawley JN (2011). Sociability and motor functions in Shank1 mutant mice. Brain Res.

[47] Song Q, Sheng W, Zhang X, Jiao S, Li F (2014). ILEI drives epithelial to mesenchymal transition and metastatic progression in the lung cancer cell line A549. Tumour Biol.

[48] Timmerman V, Clowes VE, Reid E (2013). Overlapping molecular pathological themes link Charcot–Marie–Tooth neuropathies and hereditary spastic paraplegias. Exp Neurol.

[49] Timmerman V, Strickland AV, Züchner S (2014). Genetics of Charcot-Marie-Tooth (CMT) disease within the frame of the human genome project success. Genes (Basel).

[50] Totaro A, Renzi F, La Fata G, Mattioli C, Raabe M, Urlaub H (2011). The human Pat1b protein: a novel mRNA deadenylation factor identified by a new immunoprecipitation technique. Nucleic Acids Res.

[51] Wang H, Vardy LA, Tan CP, Loo JM, Guo K, Li J (2010). PCBP1 Suppresses the Translation of Metastasis-Associated PRL-3 Phosphatase. Cancer Cell.

[52] Windpassinger C, Auer-Grumbach M, Irobi J, Patel H, Petek E, Hörl G (2004). Heterozygous missense mutations in BSCL2 are associated with distal hereditary motor neuropathy and Silver syndrome. Nat Genet.

[53] Wolozin B (2012). Regulated protein aggregation: stress granules and neurodegeneration. Mol Neurodegener.

[54] Xia C-H, Roberts EA, Her L-S, Liu X, Williams DS, Cleveland DW, Goldstein LSB (2003). Abnormal neurofilament transport caused by targeted disruption of neuronal kinesin heavy chain KIF5A. J Cell Biol.

[55] Yoga YMK, Traore DAK, Sidiqi M, Szeto C, Pendini NR, Barker A (2012). Contribution of the first K-homology domain of poly(C)-binding protein 1 to its affinity and specificity for C-rich oligonucleotides. Nucleic Acids Res.

[56] Yonekawa Y, Harada A, Okada Y, Funakoshi T, Kanai Y, Takei Y (1998). Defect in synaptic vesicle precursor transport and neuronal cell death in KIF1A motor protein-deficient mice. J Cell Biol.

