# Supplementary material for: Mutant HSPB1 causes loss of translational repression by binding to PCBP1, an RNA binding protein with a possible role in neurodegenerative disease
Source: Acta Neuropathol Commun. 2017 Jan 11;5:5. doi: 10.1186/s40478-016-0407-3 (PMC5225548; doi:10.1186/s40478-016-0407-3)
Supplement: Additional file 1: Figure S1. — HSPB1 and PCBP1 are ubiquitously expressed proteins. Figure S2: The expression of PCBP1 and HSPB1 increases during embryonal development. Figure S3: Patient fibroblasts show no protein aggregation. Figure S4: PCBP1 shows no abnormal localization in stable neuroblastoma cell lines. Figure S5: PCBP1 mRNA targets are enriched in neuronal processes. (DOCX 2584 kb) [file 40478_2016_407_MOESM1_ESM.docx]

**Supplementary Data**

**
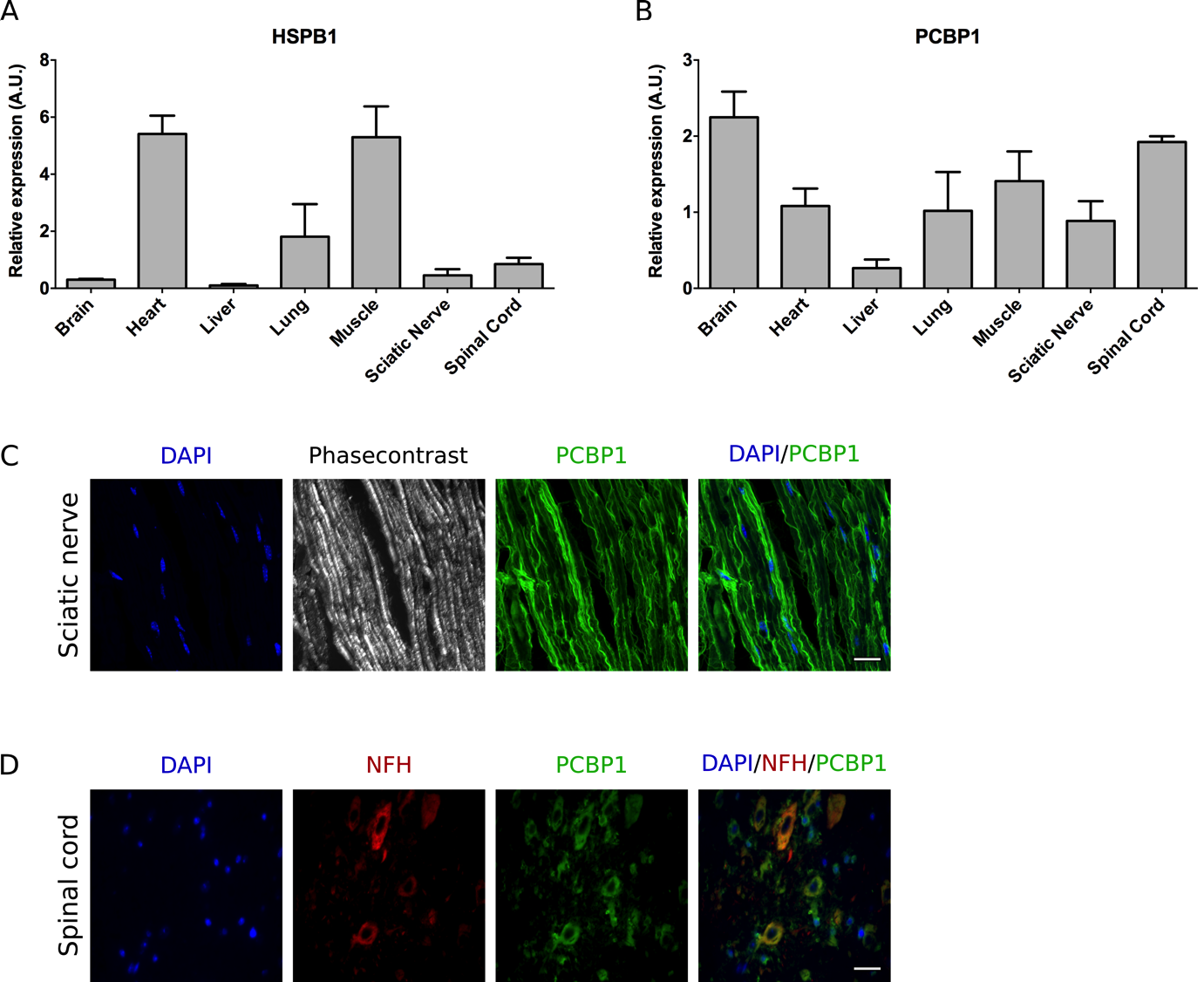
**

**Figure S1: HSPB1 and PCBP1 are ubiquitously expressed proteins**

RT-qPCR performed on different mouse tissues for HSPB1 **(A)** and PCBP1 **(B)**. Expression values of the mentioned genes were normalized to at least 4 housekeeping genes. Represented values are averages with SD as error bar. Immunohistochemistry for PCBP1 performed on mouse sciatic nerve (**C**) and spinal cord (**D**). Scale bar = 30μm

**
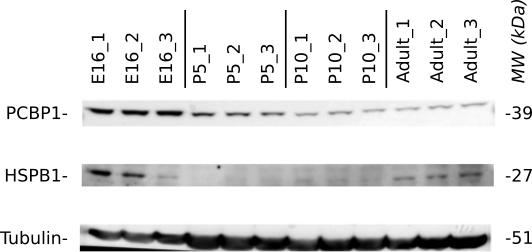
**

**Figure S2: The expression of PCBP1 and HSPB1 increases during embryonal development**

Western blot showing the expression levels of PCBP1 and HSPB1 in protein lysates originating from; embryos at day 15, pups at day 5 and 10 and 12 week old adult mice. This experiment was performed in triplicate for all different ages.

**
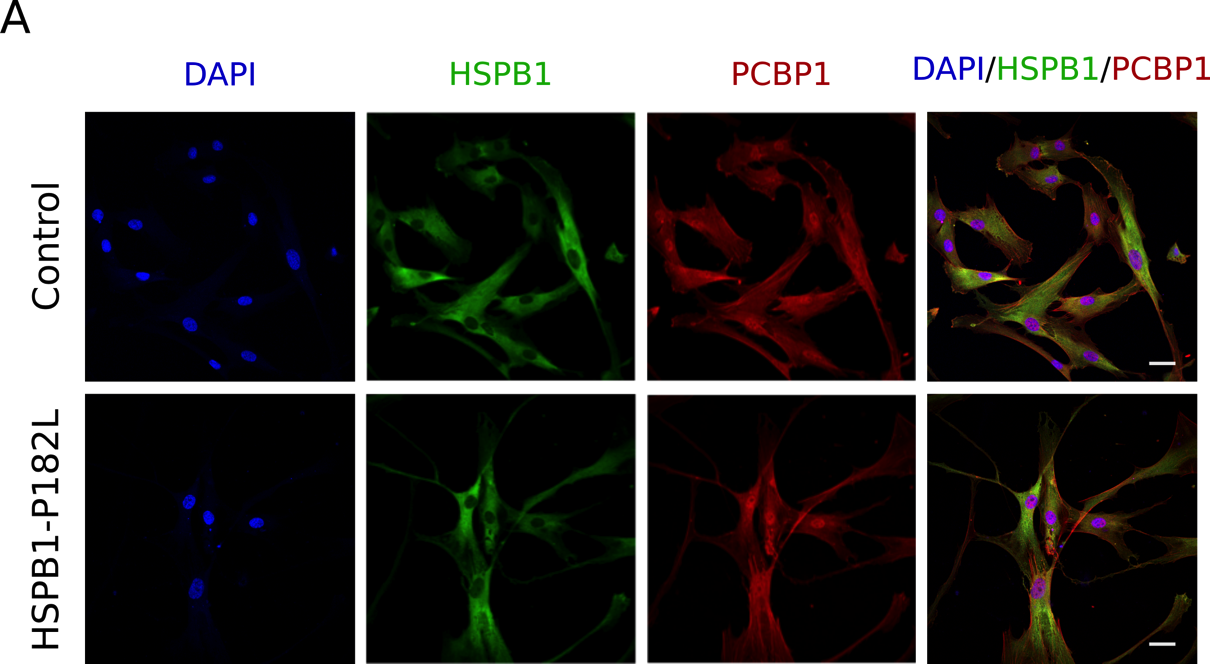
**

**Figure S3: Patient fibroblasts show no protein aggregation**

Fibroblasts derived from a healthy control individual and a dHMN patient carrying the HSPB1-P182L mutation were stained for endogenous HSPB1 and PCBP1. Scale bar = 30μm

**
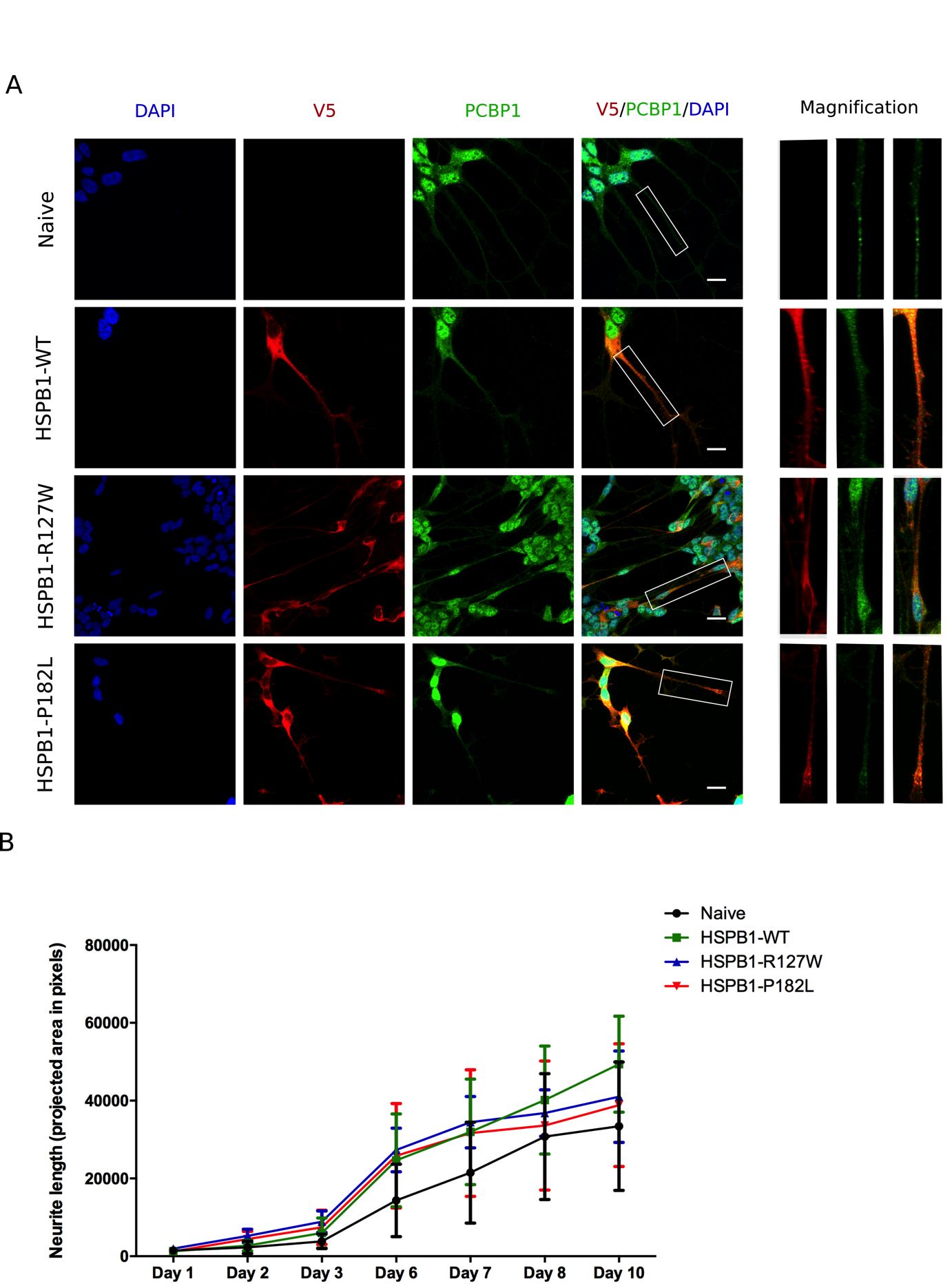
**

**Figure S4: PCBP1 shows no abnormal localization in stable neuroblastoma cell lines**

(A) SH-SY5Y cell lines were used for lentiviral transduction with HSPB1 wild type (WT) and mutants (R127W and P182L). After differentiation, these stable cell lines were used for immunocytochemistry. Wild type and mutant HSPB1 were fused with a V5-tag in order to stain specifically for the exogenous protein. Scale bar = 30μm (B) The differentiation process of stable SH-SY5Y cell lines was followed for 10 days by taking at fixed time points phase contrast images of the same microscopic field. Total neurite length was measured on each image. The graph represents mean values with SD as error bars (n=20 microscopic fields).

**
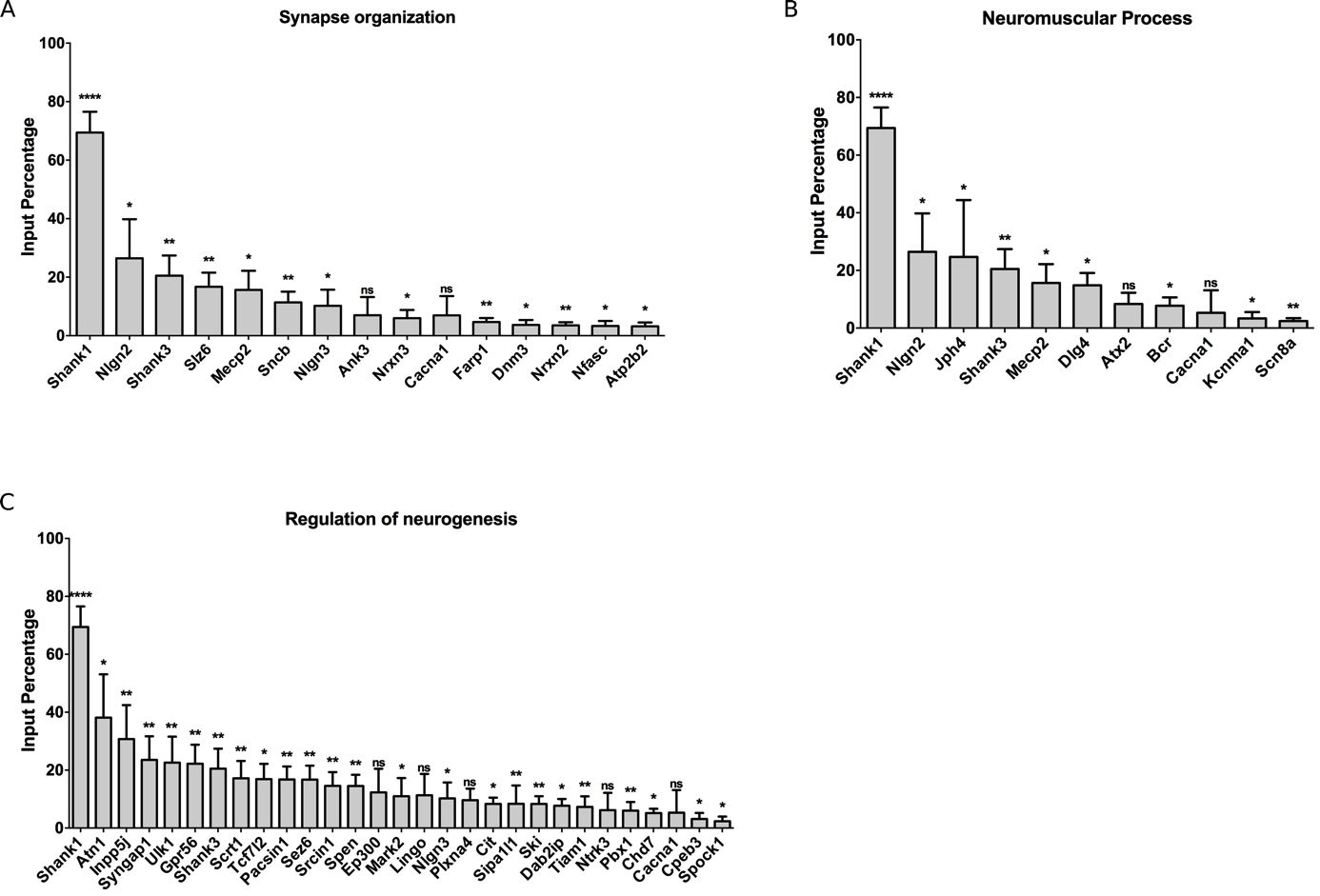
**

**Figure S5: PCBP1 mRNA targets are enriched in neuronal processes**

**(A-C)** Significantly enriched GO term categories were selected based on the data list obtained by RIP sequencing (≥ 2fold). All genes present in these GO categories were validated by RT-qPCR on normal adult mouse brain and are shown in separate graphs. All experiments were performed in triplicate and data are shown as mean with SD as error bar and are analyzed as described in the legend of Figure 4C. Multiple t-test with Holm-Sidak as a correction for multiple comparisons was used as statistical test.
