# Supplementary material for: Mutant HSPB1 causes loss of translational repression by binding to PCBP1, an RNA binding protein with a possible role in neurodegenerative disease
Source: Acta Neuropathol Commun. 2017 Jan 11;5:5. doi: 10.1186/s40478-016-0407-3 (PMC5225548; doi:10.1186/s40478-016-0407-3)
Supplement: Additional file 2: Table S1. — Full list of PCBP1 target mRNAs validated by RT-qPCR for enrichment in the PCBP1 IP (Relates to Fig. 4). (DOC 52 kb) [file 40478_2016_407_MOESM2_ESM.doc]

| **Table S1: Full list of PCBP1 target mRNAs validated by RT-qPCR for enrichment in the PCBP1 IP** (Relates to Figure 4) | | |
| --- | --- | --- |
| **Gene Symbol** | **RNASeq PCBP1 IP/Input** | **RT-qPCR PCBP1 IP/Input** |
| Ank3 | 3,176 | 0,604 |
| Atf5 | 3,009 | 9,602 |
| Atn1 | 6,287 | 8,284 |
| Atp2b2 | 3,065 | 0,64 |
| Bcl9l | 6,527 | 36,02 |
| Bcr | 3,215 | 1,388 |
| Bscl2 | 2,071 | 3,722 |
| Cacna1 | 3,156 | 1,355 |
| Chd7 | 5,217 | 1,111 |
| Cit | 5,294 | 1,379 |
| Cpeb3 | 3,585 | 0,573 |
| Dab2ip | 3,652 | 1,592 |
| Dlg4 | 4,302 | 3,343 |
| Dnm3 | 3,562 | 0,712 |
| Ep300 | 5,512 | 3,358 |
| Fa2h | 3,01 | 2,078 |
| Fam102a | 2,997 | 4,028 |
| Farp1 | 3,405 | 0,867 |
| Fmn2 | 7,53 | 18,123 |
| Gpr56 | 4,313 | 2,975 |
| Inf2 | 4,246 | 21,418 |
| Inpp5j | 3,997 | 5,257 |
| Jph4 | 4,435 | 9,696 |
| Kcnma1 | 4,547 | 0,711 |
| Kif1a | 2,311 | 1,294 |
| Kif1b | 1,985 | 1,621 |
| Kif5a | 3,089 | 0,779 |
| Lasp1 | 2,996 | 2,715 |
| Lingo | 3,397 | 1,946 |
| Mark2 | 3,626 | 1,851 |
| Mecp2 | 5,167 | 2,182 |
| Med25 | 3,056 | 4,055 |
| Mll2 | 8,377 | 22,92 |
| Nfasc | 3,017 | 0,597 |
| Nlgn2 | 5,388 | 6,042 |
| Nlgn3 | 5,085 | 1,485 |
| Nrxn2 | 4,357 | 0,735 |
| Nrxn3 | 3,747 | 1,179 |
| Ntrk3 | 4,317 | 1,805 |
| Pabpc1 | 2,806 | 1,625 |
| Pacsin1 | 4,758 | 3,437 |
| Pbx1 | 3,183 | 1,094 |
| Pclo | 6,563 | 23,412 |
| Phlpp2 | 3,003 | 2,228 |
| Pla2g3 | 3,001 | 1,125 |
|  |  |  |
| **Gene Symbol** | **RNASeq PCBP1 IP/Input** | **RT-qPCR PCBP1 IP/Input** |
| Plekhg5 | 1,546 | 1,527 |
| Plxna4 | 5,232 | 1,88 |
| Scn8a | 3,296 | 0,471 |
| Scn9q | 1,015 | 0,175 |
| Scrt1 | 4,099 | 4,712 |
| Sez6 | 3,69 | 3,624 |
| Shank1 | 8,064 | 54,272 |
| Shank3 | 5,27 | 4,171 |
| Sipa1l1 | 4,463 | 1,873 |
| Ski | 3,427 | 1,843 |
| Slc12a6 | 1,136 | 2,553 |
| Sncb | 3,817 | 2,062 |
| Sox10 | 2,633 | 10,622 |
| Spen | 6,37 | 3,785 |
| Spock1 | 3,074 | 0,35 |
| Srcap | 7,38 | 21,409 |
| Srcin1 | 4,462 | 2,126 |
| Syngap1 | 5,832 | 4,155 |
| Tcf7l2 | 4,419 | 3,81 |
| Tiam1 | 3,154 | 1,334 |
| Tulp4 | 2,996 | 1,012 |
| Ulk1 | 5,377 | 3,107 |
| Wnk1 | 4,293 | 8,391 |
| Zcchc2 | 3 | 3,217 |
| Zmiz1 | 6,561 | 15,882 |
| 2210408I21Rik | 2,994 | 1,12 |
| 2310061I04Rik | 3,01 | 1,259 |
