# Supplementary material for: Mutant HSPB1 causes loss of translational repression by binding to PCBP1, an RNA binding protein with a possible role in neurodegenerative disease
Source: Acta Neuropathol Commun. 2017 Jan 11;5:5. doi: 10.1186/s40478-016-0407-3 (PMC5225548; doi:10.1186/s40478-016-0407-3)
Supplement: Additional file 4: Table S3. — PCBP1 mRNA targets used for mouse UTR database search. Relates to Fig. 5. (DOC 80 kb) [file 40478_2016_407_MOESM4_ESM.doc]

| **Table S3: PCBP1 mRNA targets used for mouse UTR database search** | | | | |  |  |
| --- | --- | --- | --- | --- | --- | --- |
| Relates to Figure 5 | |  |  |  |  |  |
|  |  |  |  |  |  |  |
|  |  |  |  |  |  |  |
| **High enriched targets** | | |  | **Less enriched targets** | | |
|  |  |  |  |  |  |  |
| **Gene** | **5'UTR (bp)** | **3'UTR (bp)** |  | **Gene** | **5'UTR (bp)** | **3'UTR (bp)** |
| Mll2 | 207 | 2831 |  | Mcart6 | 596 | 1554 |
| Shank1 | 76 | 1016 |  | Matr3 | 316 | 1615 |
| Fmn2 | 221 | 1484 |  | Atp5f1 | 562 | 123 |
| Pclo | 304 | 4573 |  | Ttr | 189 | 588 |
| Zmiz1 | 499 | 1672 |  | Atp6v1a | 72 | 2020 |
| Bcl9l | 214 | 640 |  | Napb | 57 | 2739 |
| 9830001H06Rik | 356 | 7911 |  | Spsb2 | 169 | 215 |
| Prr12 | 185 | 771 |  | Cnp | 127 | 943 |
| Spen | 351 | 1016 |  | Skp1a | 124 | 838 |
| Zfhx2 | 90 | 946 |  | Sdha | 32 | 832 |
| Atn1 | 340 | 528 |  | Hnrnpk | 151 | 435 |
| Nova2 | 151 | 7064 |  | Pfkm | 149 | 388 |
| Raph1 | 201 | 51 |  | Hnrnpa2b1 | 218 | 2842 |
| Ubn2 | 248 | 10476 |  | Got1 | 150 | 666 |
| Ncor2 | 370 | 3402 |  | Snhg11 | 370 | 5230 |
| Map3k9 | 296 | 1273 |  | Slc25a3 | 52 | 386 |
| Arid1a | 276 | 1323 |  | Ndfip1 | 139 | 132 |
| Pvrl1 | 68 | 3445 |  | Sept7 | 71 | 1013 |
| Nfix | 316 | 1099 |  | Pja2 | 175 | 2407 |
| Pom121 | 41 | 1584 |  | Prnp | 184 | 1237 |
| Brd4 | 255 | 1478 |  | Ghitm | 111 | 1324 |
| Tet3 | 231 | 5669 |  | Cox4i1 | 67 | 102 |
| Crebbp | 181 | 3589 |  | Zwint | 24 | 1893 |
| Wipf2 | 373 | 5893 |  | Rpl4 | 44 | 118 |
| Shisa7 | 247 | 4083 |  | Cst3 | 91 | 228 |
| Zfp609 | 226 | 4117 |  | Sqstm1 | 33 | 638 |
| BC068157 | 343 | 976 |  | Itm2b | 145 | 827 |
| Shank2 | 584 | 1540 |  | Serinc1 | 134 | 1383 |
| Nav1 | 35 | 7104 |  | Tmsb4x | 128 | 407 |
| Slc25a23 | 190 | 1768 |  | Apoe | 179 | 136 |
| Rnf165 | 34 | 6222 |  | Nap1l5 | 159 | 1264 |
| 1700008O03Rik | 176 | 143 |  | Ddx5 | 178 | 1494 |
| D10Bwg1379e | 133 | 1570 |  | Eif4a2 | 458 | 821 |
| Kcnc3 | 365 | 2746 |  | Tubb2a | 79 | 190 |
| Srgap1 | 456 | 156 |  | Klc1 | 195 | 458 |
| Cic | 671 | 613 |  | Ppp3r1 | 242 | 2074 |
| Egr3 | 273 | 252 |  | Ldhb | 97 | 188 |
| Rere | 304 | 2477 |  | Sparcl1 | 723 | 441 |
| Nav2 | 752 | 3267 |  | Slc25a4 | 87 | 124 |
|  |  |  |  |  |  |  |
| **High enriched targets** | | |  | **Less enriched targets** | | |
|  |  |  |  |  |  |  |
| **Gene** | **5'UTR (bp)** | **3'UTR (bp)** |  | **Gene** | **5'UTR (bp)** | **3'UTR (bp)** |
| Bcl9 | 142 | 232 |  | Aldoc | 204 | 406 |
| Ttyh3 | 203 | 2879 |  | Cpe | 91 | 581 |
| Camta2 | 284 | 736 |  | Mdh1 | 202 | 751 |
| Gm608 | 299 | 5641 |  | Atp5a1 | 283 | 487 |
| Nptxr | 126 | 3341 |  | Actg1 | 66 | 700 |
| Shisa6 | 162 | 5747 |  | Hsp90aa1 | 144 | 504 |
| Auts2 | 520 | 1754 |  | Atp5b | 20 | 269 |
| Atxn2l | 110 | 458 |  | Ckb | 124 | 208 |
| Cd47 | 133 | 811 |  | Calm2 | 140 | 626 |
| Ep300 | 415 | 1095 |  | Ubb | 98 | 149 |
| Gm98 | 144 | 2114 |  | Snap25 | 213 | 1285 |
| Wbp7 | 9 | 306 |  |  |  |  |
| Nhsl2 | 183 | 9128 |  |  |  |  |
| Tnrc6b | 290 | 11610 |  |  |  |  |
